# Developmental regulation of regenerative potential in *Drosophila* by ecdysone through a bistable loop of ZBTB transcription factors

**DOI:** 10.1371/journal.pbio.3000149

**Published:** 2019-02-11

**Authors:** Karine Narbonne-Reveau, Cédric Maurange

**Affiliations:** Aix Marseille Université, CNRS, IBDM, UMR 7288, Marseille, France; New York University, UNITED STATES

## Abstract

In many organisms, the regenerative capacity of tissues progressively decreases as development progresses. However, the developmental mechanisms that restrict regenerative potential remain unclear. In *Drosophila*, wing imaginal discs become unable to regenerate upon damage during the third larval stage (L3). Here, we show that production of ecdysone after larvae reach their critical weight (CW) terminates the window of regenerative potential by acting on a bistable loop composed of two antagonistic Broad-complex/Tramtrack/Bric-à-brac Zinc-finger (ZBTB) genes: *chinmo* and *broad* (*br*). Around mid L3, ecdysone signaling silences *chinmo* and activates *br* to switch wing epithelial progenitors from a default self-renewing to a differentiation-prone state. Before mid L3, Chinmo promotes a strong regenerative response upon tissue damage. After mid L3, Br installs a nonpermissive state that represses regeneration. Transient down-regulation of ecdysone signaling or Br in late L3 larvae enhances *chinmo* expression in damaged cells that regain the capacity to regenerate. This work unveils a mechanism that ties the self-renewing and regenerative potential of epithelial progenitors to developmental progression.

## Abstract 2

This study finds that the loss of regeneration potential in Drosophila wing imaginal discs is induced by the production of the steroid hormone ecdysone after the larva reaches its critical weight. Manipulating ecdysone signaling or the downstream transcription factors can uncouple regenerative properties from developmental progression.

## Author summary

While some organisms exhibit remarkable regenerative abilities throughout their life, many animals, including mammals, present limited regenerative potential that progressively decreases during development. Understanding the mechanisms underlying this progressive loss is important to devise therapeutic approaches aiming at facilitating the regeneration of a damaged tissue throughout life. The fruitfly *Drosophila* is a powerful model organism to address such questions. Indeed, while tissues, such as imaginal discs, can fully regenerate if damaged during early development, they fail to do so upon damages during late development. We show here that restriction of regenerative potential occurring during midlarval stages is due to the production of a steroid hormone, named ecdysone. By genetically manipulating ecdysone signaling, we can uncouple regenerative abilities from developmental progression. In particular, we show that ecdysone signaling triggers a switch in the sequential expression of two transcription factors, Chinmo and Broad, that positively and negatively regulate the competence for imaginal disc regeneration, respectively. Our work therefore identifies a key developmental signal that restricts regenerative potential in insects and opens new perspectives on elucidating how regeneration-permissive transcriptional programs are locked as development progresses.

## Introduction

The impressive ability of some animals to regenerate damaged tissues has fascinated biologists for centuries. However, in many animals, including humans, most tissues lose the ability to regenerate as development progresses [1]. In mice, digit or heart regenerative capacities follow a gradual decline from fetal to early postnatal stages [2–4]. In the frog *Xenopus laevis*, the ability to efficiently regenerate limbs upon amputation is lost during metamorphosis [5]. Yet, the molecular mechanisms that progressively restrict the regenerative potential of tissues during development remain elusive.

*Drosophila* is a powerful model to investigate this question. Imaginal discs are epithelial sacs that form at the end of embryogenesis, undergo rapid growth during larval stages, and differentiate during metamorphosis to generate various adult structures. Pioneer work in the 1940s showed that imaginal discs exhibit the ability to regenerate if manually damaged [6]. Since then, elegant genetic systems have made possible the in vivo ablation of specific regions of the wing imaginal disc to investigate regeneration in great details [7,8]. For example, it has been shown that ablation of the wing pouch by the transient expression of proapoptotic genes triggers the production of reactive oxygen species (ROS) and activation of the c-Jun N-terminal kinase (JNK) pathway. If ablation is performed during early third larval stage (early L3), ROS and JNK pathway activity elicit activation of the Janus Kinase/Signal Transducers and Activators of Transcription (JAK/STAT) and Wingless (Wg) signaling, leading to a cascade of events triggering regenerative growth and a normal wing in adults. In contrast, the regenerative JAK/STAT- and Wg-mediated response fail to be efficiently activated if the wing pouch is ablated after mid L3, leading to an absence of wings in adults [9–12]. Thus, the regenerative capacity of imaginal discs is limited to an early developmental window that progressively terminates as larvae progress to the end of L3 (late L3). Interestingly, chromatin rearrangements between early L3 and late L3 at the *wg* locus appear to restrict the accessibility of the gene to transcription factors, making it less susceptible for activation upon damages in late larvae [13]. However, the temporal signals that instruct chromatin rearrangements to restrain the regenerative ability of imaginal discs as development proceeds are still unclear but may be linked to the approach of metamorphosis. Irreversible commitment to metamorphosis is triggered by an important developmental milestone known as the critical weight (CW) that, in *Drosophila melanogaster*, is usually reached about 8 to 12 hours after the L2/L3 molt under rich food condition at 25°C [14–16]. By a still unclear mechanism, the reaching of the CW leads to the production and release of increasing levels of the steroid hormone ecdysone by the prothoracic gland. Ecdysone and its mature form, 20-hydroxyecdysone (20-HE), trigger a cascade of events with pleiotropic effects in the various larval tissues, allowing the progressive deployment of metamorphosis programs [17]. Yet, the progressive molecular changes triggered in imaginal discs by increasing levels of ecdysone after the CW are still not fully deciphered. Interestingly, ectopic feeding of early larvae with ecdysone precociously restricts regenerative capacity [18,19], while preventing ecdysteroid synthesis appears to prolong the capacity to initiate efficient regeneration [20]. However, in this process, it is still unknown whether ecdysone acts cell-autonomously on wing epithelial tissues or non-cell-autonomously via intermediate signals.

The *broad* (*br*) gene is an early target of ecdysone signaling in wing imaginal discs. *br* codes for four protein isoforms (Br-Z1 to Br-Z4) of the Broad-complex/Tramtrack/Bric-à-brac Zinc-finger (ZBTB) transcription factor family [21]. All isoforms share a common core amino terminus fused to any one of four pairs of C2H2-type Zinc-finger domains [21–24]. *br* is activated by ecdysone in wing imaginal discs a few hours after the CW has been reached [25–27]. In wing discs, the Br proteins are required to promote the specification and differentiation of epithelial cells into sensory organ precursors [26]. However, the different roles of the different Br isoforms remain poorly understood. Another ZBTB transcription factor, Chinmo, is silenced after the CW by ecdysone in the neuroepithelium of the developing optic lobe in the *Drosophila* brain [28]. While Br appears to promote differentiation in wing discs, Chinmo appears to maintain an undifferentiated state in the neuroepithelium. *chinmo* is also known to be expressed in eye imaginal discs [29], but its expression pattern, mode of regulation, and role in wing discs is less clear.

Here, we find that Chinmo and Br are sequentially expressed during wing disc development and exhibit cross-repressive activities to define a bistable loop. By switching wing epithelial progenitors from a Chinmo^+^ to a Br^+^ state, activation of ecdysone signaling after the CW terminates a default self-renewing state, activates differentiation, and restricts regenerative potential. Importantly, manipulation of the bistable loop can restore effective regeneration in late L3 larvae, therefore uncoupling regenerative abilities from developmental progression.

## Results

### Chinmo is required for efficient wing imaginal disc regeneration

**Fig 1:**
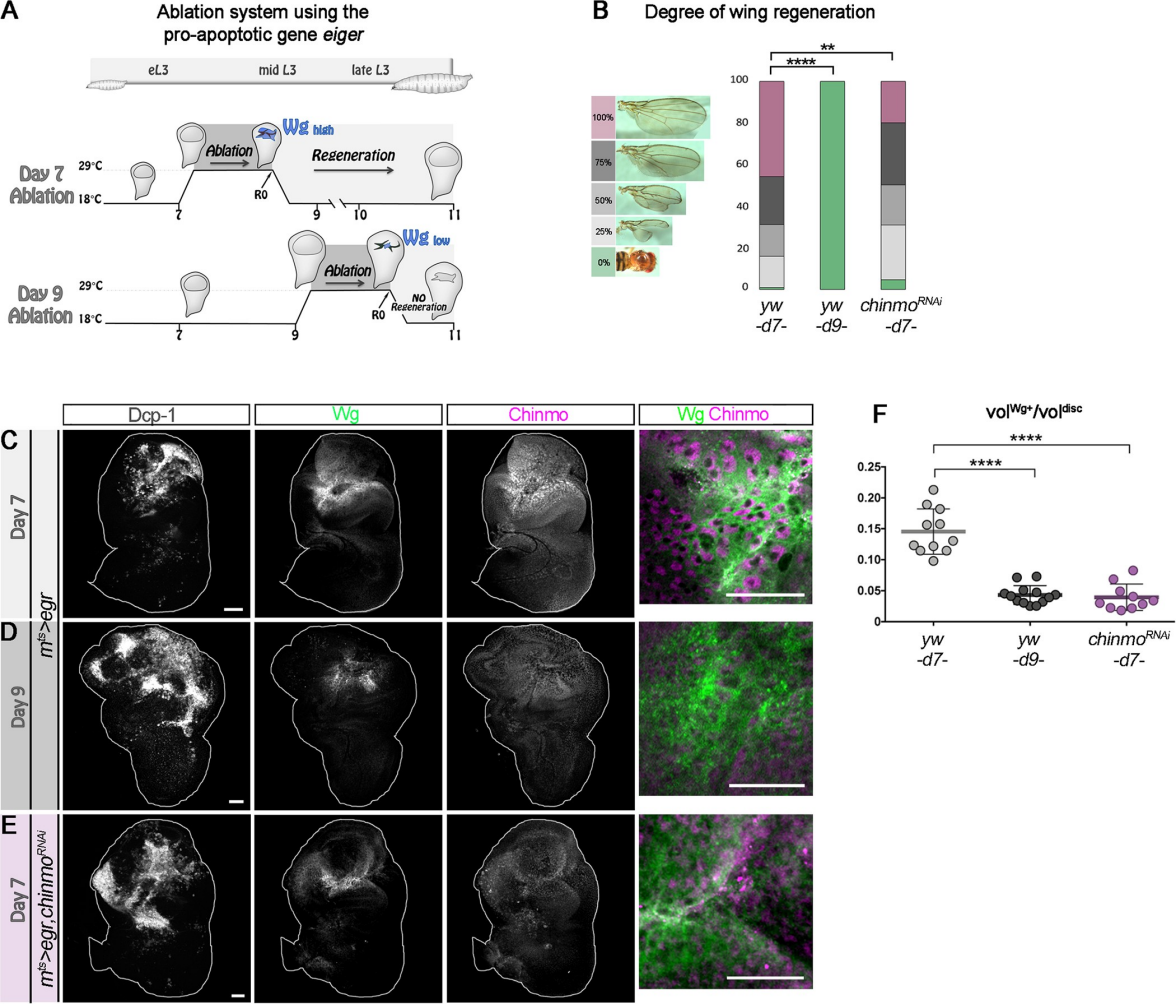
Chinmo is up-regulated in the blastema of damaged wing imaginal discs and promotes efficient regeneration. (A) Schematic representation of the *rn*^*ts*^*>egr* ablation system used to induce wing pouch ablation. Strong *wg* expression at R0 is observed in response to damage when ablation is initiated at d7 for 40 hours. *wg* expression is drastically reduced when ablation is initiated at d9. From [9]. (B) Distribution of degrees of wing regeneration in *rn*^*ts*^*>egr*,*yw* adults after d7 ablation (*n* = 617 wings), *rn*^*ts*^*>egr*,*yw* adults after d9 ablation (*n* = 216 wings), and *rn*^*ts*^*>egr*,*chinmo*^*RNAi*^ adults after d7 ablation (*n* = 344 wings). *p* = 2.2 × 10^−57^ and *p* = 0.0013 (*rn*^*ts*^*>egr*,*yw* at d7 compared to *rn*^*ts*^*>egr*,*yw* at d9 and *rn*^*ts*^*>egr yw* at d7 compared to *rn*^*ts*^*>egr*,*chinmo*^*RNAi*^ at d7, respectively). (C) Anti-Dcp-1 (gray), anti-Wg (green), and anti-Chinmo (magenta) stainings at R0 in an *rn*^*ts*^*>egr* wing disc after d7 ablation. Blow-up shows that *wg* and *chinmo* are highly coexpressed in wing pouch cells. (D) Anti-Dcp-1 (gray), anti-Wg (green), and anti-Chinmo (magenta) stainings at R0 in an *rn*^*ts*^*>egr* wing disc after d9 ablation. Blow-up shows that Wg and Chinmo are low in the wing pouch. (E) Anti-Dcp-1 (gray), anti-Wg (green), and anti-Chinmo (magenta) stainings at R0 in a *rn*^*ts*^*>egr*,*chinmo*^*RNAi*^ wing disc after d7 ablation. Blow-up shows that both Wg and Chinmo are low in wing pouch cells. (F) Volume of anti-Wg staining over total wing disc volume at R0 upon d7 ablation in *rn*^*ts*^*>egr* larvae (*n* = 11 wing discs, m = 0.146 ± 0.011), upon d9 ablation in *rn*^*ts*^*>egr* larvae (*n* = 13 wing discs, m = 0.043± 0.004), and upon d7 ablation in *rn*^*ts*^*>egr*,*chinmo*^*RNAi*^ larvae (*n* = 10 wing discs, m = 0.039 ± 0.007). *p* = 8.0 × 10^−7^ and *p* = 5.7 × 10^−6^ (*rn*^*ts*^*>egr*,*yw* at d7 compared to *rn*^*ts*^*>egr*,*yw* at d9 and *rn*^*ts*^*>egr*,*yw* at d7 compared to *rn*^*ts*^*>egr*,*chinmo*^*RNAi*^ at d7, respectively). Scale bars: 30 μm. Underlying data for Fig 1 can be found in S1 Data. d, day; Dcp-1, Death Caspase-1; *egr*, *eiger*; RNAi, RNA interference; *rn*^*ts*^, *rotund-GAL4*, *tubulin-GAL80*^*thermo-sensitive*^; R0, beginning of the recovery period; vol, volume; Wg, Wingless; *yw*, *yellow*,*white*.

We have previously demonstrated that Chinmo promotes self-renewal in neuroblasts (NBs) of the ventral nerve cord and central brain and in the neuroepithelium of the optic lobe during early larval stages [28,30]. Interestingly, a transcriptomic analysis has recently identified *chinmo* to be up-regulated in the blastema of regenerating wing discs [31]. This prompted us to investigate the function and cause of this up-regulation. For this purpose, we used the elegant protocol devised by Smith-Bolton and colleagues [9] that combines the Gal4/UAS/GAL80^ts^ systems to genetically trigger wing pouch ablation at defined stages of larval development through the transient misexpression of the proapoptotic gene *eiger* (*egr*) via a temporally controlled temperature switch from 18°C to 29°C for 40 hours (Fig 1A) [9]. Using this *rn*^*ts*^*>egr* system (*rotund-GAL4*,*tubulin-GAL80*^*thermo-sensitive*^[*rn*^*ts*^]*;UAS-eiger*), previous work has shown that wing pouch ablation in early L3 larvae (day 7 [d7]) triggers high expression of *wg* (Fig 1A, 1C and 1F and S1A and S1B Fig) and efficient regeneration as assessed by the size and shape of the wing in the adult (Fig 1B) [9]. In contrast, wing pouch ablation in mid/late L3 larvae (day 9 [d9]) leads to a more restricted and less intense activation of *wg* and is unable to induce efficient regeneration (Fig 1A, 1B, 1D and 1F and S1B Fig) [9].

In agreement with the previous transcriptomic study, we find that wing pouch ablation at d7 triggered high levels of Chinmo in Wg^+^ cells of the regenerating blastema after 40 hours of *egr* misexpression (R0), while Chinmo levels were significant but lower in cells surrounding the blastema (Fig 1C and S1C Fig). *wg* has been shown to be strongly expressed in the blastema cells but not in the apoptotic tissue [9]. Accordingly, we see no Chinmo in apoptotic cells expressing the effector caspase Dcp-1 (S1D Fig). This protocol led to full wing regeneration in almost half of the adults, as indicated by normal-sized wings (Fig 1B). In contrast, wing pouch ablation at d9 using the same protocol triggered a more restricted expression pattern of both *wg* and *chinmo* at R0 and resulted in an absence of regenerated wing in the adult (Fig 1B, 1D and 1F and S1B and S1C Fig). When *chinmo* was knocked down during the d7 ablation protocol through concomitant expression of a *UAS-chinmo*^*RNAi*^ transgene, we observed that the levels of Wg significantly decreased (Fig 1E and 1F and S1B and S1C Fig). In addition, transient *chinmo* knockdown reduced full regeneration from half to about a fifth of the adult flies (Fig 1B). In this condition, the number of flies exhibiting no or low levels of regeneration also increased compared to control (Fig 1B). A similar decrease in regeneration efficiency was observed when the pro-apoptotic gene *reaper* (*rpr*) was used instead of *egr* (S1E and S1F Fig) to induce d7 wing pouch ablation. Together, these experiments suggest that Chinmo is cell-autonomously required for the efficient expression of *wg* in the blastema after early larval ablation of the wing pouch and facilitates efficient wing regeneration.

### The ZBTB genes *chinmo* and *br-Z1* are sequentially expressed in wing disc epithelia

**Fig 2:**
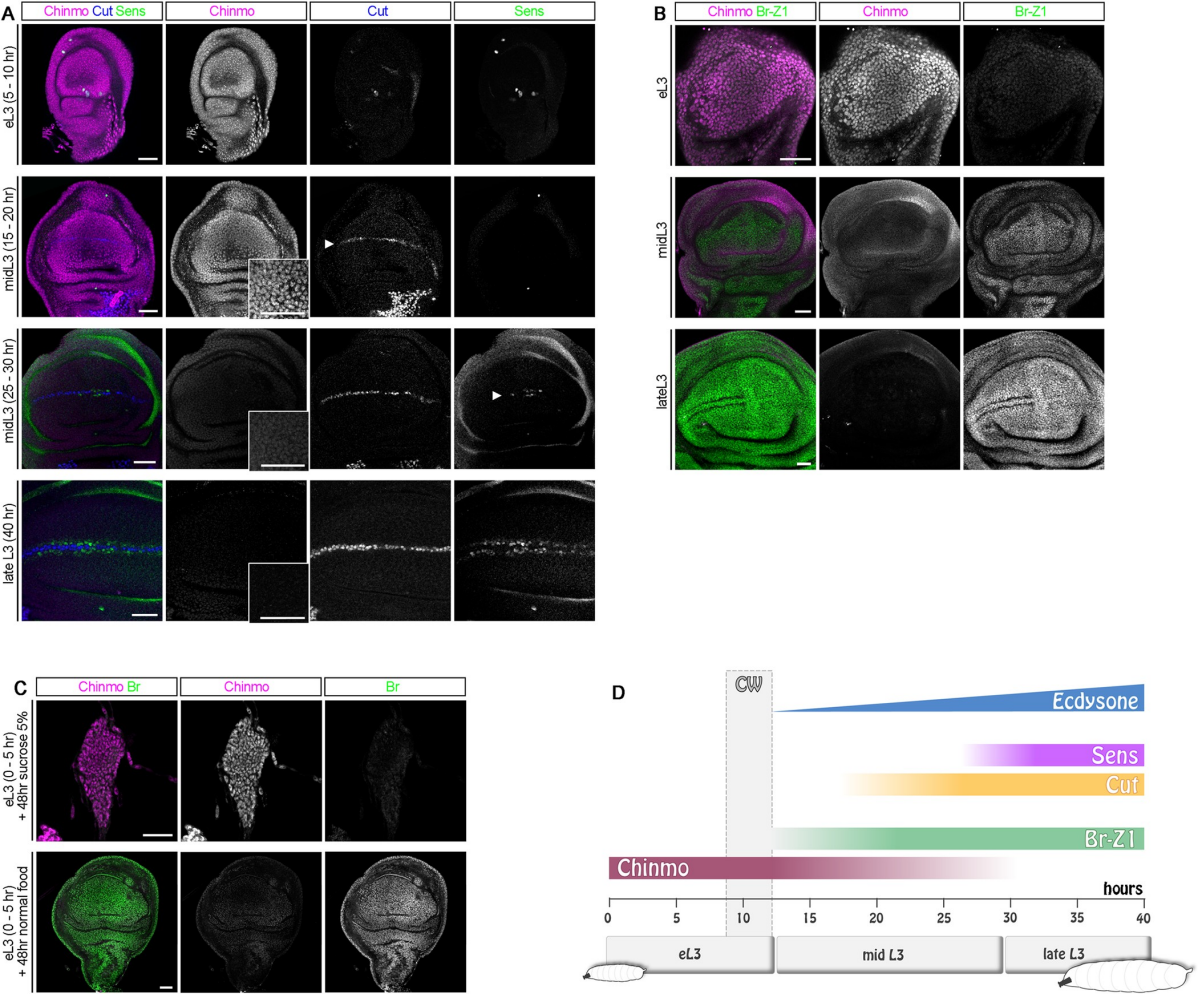
Chinmo down-regulation and Br up-regulation occur shortly after the CW has been reached. (A) Chinmo (magenta) protein level is high in the wing disc during early L3 (5–10 hours) but rapidly decreases shortly after the CW when *cut* starts to be expressed (arrowhead, mid L3, 15–20 hours). Chinmo is absent when Sens starts to be expressed (arrowhead, mid L3, 25–30 hours) and remains absent in late L3 stage (late L3, 40 hours). (B) Chinmo (magenta) and Br-Z1 (green) protein levels in the wing disc during early L3, after the CW (mid L3), and in late L3. (C) Wing imaginal discs of early L3 larvae transferred in sucrose 5% for 48 hours before the CW maintain high levels of Chinmo and no Br. In contrast, discs of control larvae of the same age maintained on normal food exhibit an absence of Chinmo but high Br. (D) Schematic outline of the above experiment. Scale bars: 30 μm. *br*, *broad*; CW, critical weight; eL3, early L3; hr, hours; L3, last larval stage; *sens*, *senseless*.

We then sought to understand why *chinmo* was specifically expressed in the blastema in the d7 protocol but to a lesser extent in the d9 protocol. For this purpose, knowing that *chinmo* expression is dynamically regulated in the progenitors of the developing central nervous system (CNS) [28,32,33], we looked at the dynamics of *chinmo* expression in the wing disc during larval development. We found that Chinmo is present in all the cells of the wing disc epithelium (including wing pouch and notum) during early larval development and is rapidly down-regulated around mid L3 (Fig 2A). To establish the precise time course of *chinmo* expression during larval development, we followed its expression dynamics relative to the stepwise activation of *cut* and *senseless* (*sens*) that are involved in the early stages of sensory organ specification during L3 [27,34,35]. It has been shown that a thin stripe of Cut is first detected by immunostaining at the dorsoventral boundary of the wing pouch at around 20 hours after the L2/L3 molt (at 25°C). As Cut levels progressively increase, Sens expression starts to be detected around 25–30 hours in a few cells located in the center of the pouch on both sides of the Cut stripe. At 35 hours, the Sens staining has extended all along the cut stripe [27]. Interestingly, we found that Chinmo levels are high in L3 wing imaginal discs before Cut and Sens become visible (Fig 2A, 5–10 hours). Chinmo is still present at 20 hours (Fig 2A, 15–20 hours) but is completely absent after 30 hours (Fig 2A, 40 hours). We also noted that *chinmo* down-regulation correlated with *br* activation in the wing disc throughout L3. More specifically, we identified the Br-Z1 isoform as being strongly expressed from 30 hours onwards, while Br-Z3 and Br-Z2 were absent (Fig 2B and S2A and S2B Fig). We did not test for *br-Z4* expression. *br*, *cut*, and *sens* expression in wing discs can only be activated once the larvae has reached its CW [25–27]. In *Drosophila*, the CW is reached 8 to 12 hours after the L2/L3 molt (at 25°C) on rich food (our diet) [27]. L3 larvae starved before the CW exhibit a developmental arrest and rapidly die unless they are provided with an energy source like sucrose [17,36,37]. To test whether *chinmo* silencing requires passing the CW, we transferred pre-CW L3 larvae (0–5 hours after L2/L3 molt) to 5% sucrose agar medium. In the absence of amino acids, larvae stopped growing. Interestingly, 48 hours after the transfer, imaginal discs still exhibited high levels of Chinmo and an absence of Br (Fig 2C). In contrast, control larvae kept on the standard medium continued growing, and their imaginal discs exhibited no Chinmo and strong Br (Fig 2C). Thus, *chinmo* silencing requires the larva to pass the CW. Altogether, our data indicate that during L3, wing imaginal discs sequentially express the two ZBTB transcription factors *chinmo* and *br*, and the transition from a Chinmo^+^ to a Br^+^ state requires passing the CW (Fig 2D).

### Ecdysone triggers a transcriptional Chinmo-to-Br switch

**Fig 3:**
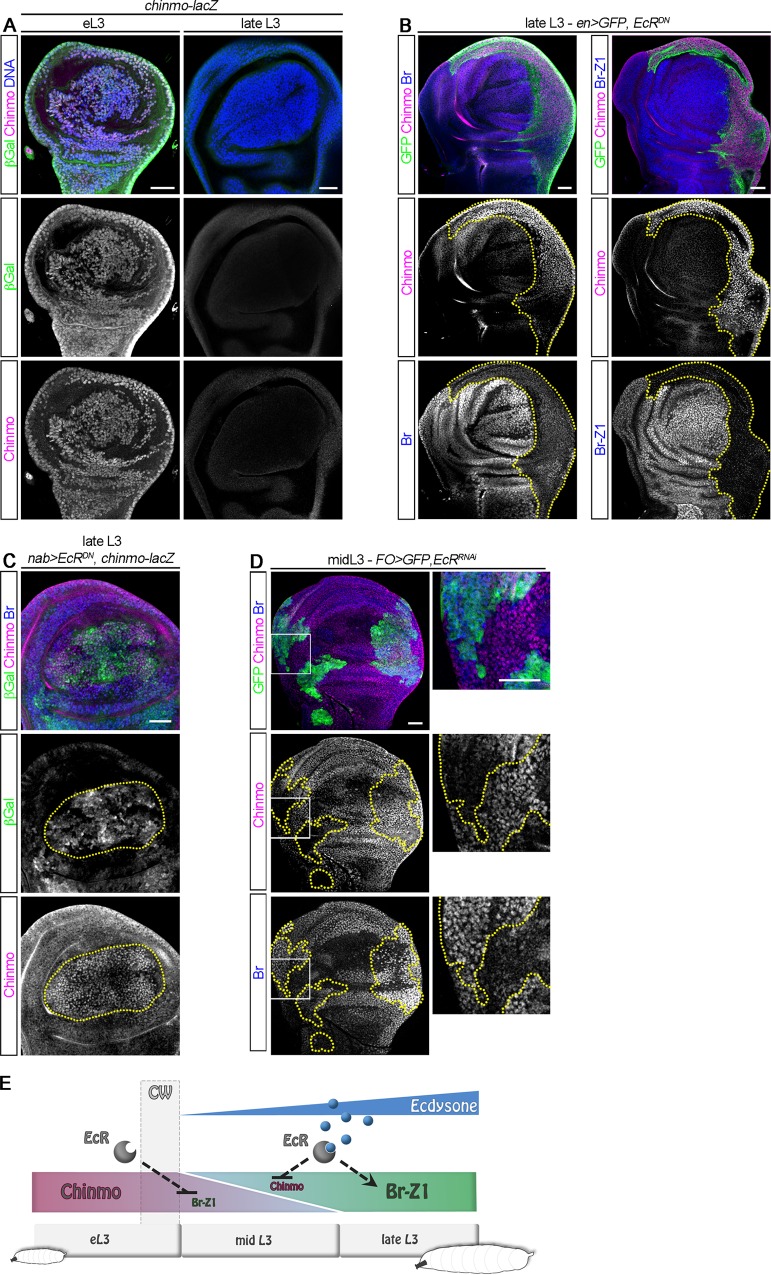
Ecdysone signaling cell-autonomously induces a Chinmo-to-Br switch. *GAL4* expression and *Flip-out* clones are marked with GFP and outlined in yellow. (A) A *chinmo-lacZ* enhancer trap exhibits transcriptional silencing in late L3 larvae. (B) Misexpression of *EcR*^*DN*^ in the posterior compartment of the wing disc using *en-GAL4* prevents *chinmo* silencing (magenta) and triggers *br* and *br-Z1* repression (blue) in late L3. (C) Misexpression of *EcR*^*DN*^ using *nab-GAL4* leads to ectopic *chinmo-lacZ* expression in the wing pouch of late L3 larvae. (D) *Flip-out* clones misexpressing *EcR*^*RNAi*^ show decreased anti-Chinmo staining (magenta, 21/22 clones, *n* = 7 discs) and increased anti-Br staining (blue, 21/22 clones, *n* = 7 discs) in mid L3. (E) Schematic outline of the above experiments. Dotted lines may represent direct or indirect regulatory interactions. Scale bars: 30 μm. *br*, *broad*; Br-Z1, Broad-Z1; β-Gal, β-Galactosidase; CW, critical weight; *EcR*^*DN*^, dominant negative form of ecdysone receptor; eL3, early L3; *en*, *engrailed*; *FO*, *Flip-out*; GFP, green fluorescent protein; L3, third larval stage; RNAi, RNA interference.

In NBs, *chinmo* is post-transcriptionally silenced via its UTRs upon progression of sequentially expressed temporal transcription factors, while it is transcriptionally silenced by ecdysone signaling in the neuroepithelium [28,38,39]. We then investigated which of these mechanisms silence *chinmo* in the wing epithelium. We first tested whether a post-transcriptional mechanism could contribute to *chinmo* silencing like in NBs. To visualize *chinmo* post-transcriptional regulation, we used (*nab-GAL4*), which is active in the wing pouch throughout development, to induce the transcription of a UAS-*mCherry*^*chinmoUTRs*^ transgene, in which the *mCherry* ORF is flanked by the 5′ and 3′ UTRs of *chinmo* [28]. We observed high levels of mCherry at all stages of larval development, showing that post-transcriptional repression of chinmo is not operating during larval stages in wing disc cells (S3A Fig), as it is in NBs. We then used a *chinmo-lacZ* enhancer trap line to assess *chinmo* transcriptional activity [28,29,32]. While *lacZ* was strongly expressed in wing disc cells early on, it became silenced around mid L3, consistent with a transcriptional silencing of *chinmo* (Fig 3A).

We next tested whether ecdysone produced after the CW could be responsible for *chinmo* transcriptional silencing. We expressed a dominant negative form of the ecdysone receptor, *EcR*^*DN*^. *EcR*^*DN*^ cannot bind its ligand 20-HE, the processed form of ecdysone [40]. Its misexpression therefore prevents activation of the ecdysone signaling pathway while leaving the repressive function of the unliganded EcR/Ultraspiracle complex unaffected [41]. Interestingly, misexpression of *EcR*^*DN*^ in the posterior compartment of the disc using *engrailed-GAL4* (*en-GAL4*), in the wing pouch using *nab-GAL4*, or in random *Flip-out* clones systematically led to the cell-autonomous maintenance of Chinmo in late L3 after the CW (Fig 3B and S3B and S3C Fig). This was associated with a complete repression of *br-Z1* and more generally of all *br* isoforms (Fig 3B; S3D and S3E Fig and [26]). In addition, *chinmo-lacZ* expression was aberrantly maintained during late L3 upon *EcR*^*DN*^ misexpression in the wing pouch using *nab-GAL4* (Fig 3C). Thus, ecdysone mediates the transcriptional repression of *chinmo*. Moreover, alleviating the repressive function of the unbound EcR/Ultraspiracle dimer by knocking down EcR with an *EcR*^*RNAi*^ transgene led to a precocious Chinmo-to-Br switch around mid L3, suggesting that the repressive function of unliganded EcR stabilizes the Chinmo^+^ state (Fig 3D, S3F Fig and [41]). *EcR* is expressed throughout larval stages in the wing disc, and in contrast to mushroom body neurons [42], its expression does not rely on Chinmo (S3G and S3H Fig). All together, these results demonstrate that activation of ecdysone signaling after the CW cell-autonomously triggers a transcriptional Chinmo-to-Br switch in wing disc cells (Fig 3E).

### Chinmo and Br form a bistable loop in the developing wing epithelium

**Fig 4:**
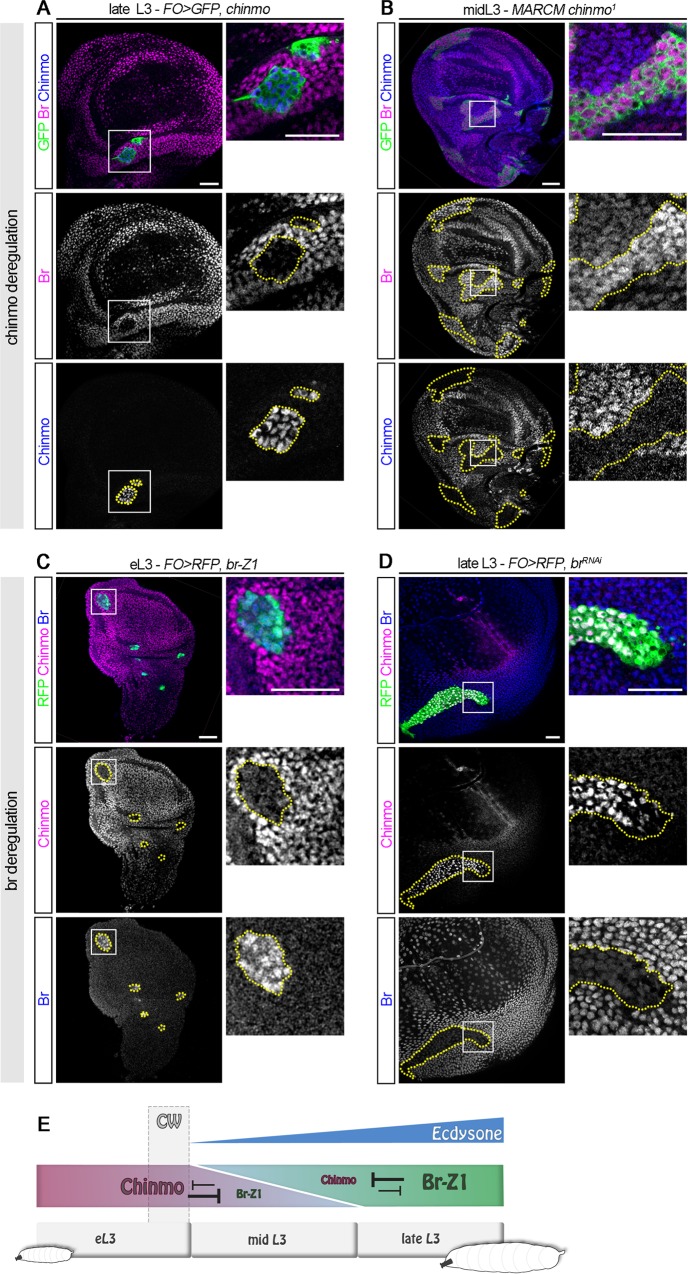
Chinmo and Br form a bistable loop during L3. *Flip-out* clones and MARCM clones are marked with GFP and outlined in yellow. (A) *Flip-out* clones misexpressing *chinmo* exhibit a down-regulation of Br (magenta, 7/7 clones, *n* = 4 discs) and ectopic Chinmo (blue) during late L3. (B) MARCM clones mutant for *chinmo* exhibit increased *br* expression (magenta, 31/31 clones, 7 discs) during eL3 stage. (C) Misexpression of *br-Z1* in *Flip-out* clones during early L3 leads to repression of *chinmo* (magenta, 16/17 clones, *n* = 8 discs). (D) Chinmo (magenta) is ectopically expressed during late L3 in *br*^*RNAi*^ *Flip-out* clones (11/11 clones, *n* = 5 discs). (E) Schematic outline of the above experiments. Scale bars: 30 **μ**m. *br*, *broad*; CW, critical weight; eL3, early L3; *FO*, *Flip-out*; GFP, green fluorescent protein; L3, third larval stage; RFP, red fluorescent protein; RNAi, RNA interference.

Important transcriptional switches during development are often stabilized by mutual repression between the involved transcription factors [43]. We thus tested whether *chinmo* and *br* cross-regulate each other. We found that misexpression of *chinmo* cell-autonomously blocked *br* activation in late L3 (Fig 4A and S4A and S4B Fig). Conversely, loss of Chinmo in *chinmo*^*1*^ mutant Mosaic Analysis with a Repressible Marker (MARCM) clones induced a precocious expression of *br* during midlarval stages (Fig 4B). In addition, precocious misexpression of *br-Z1* led to *chinmo* repression in mid L3 (Fig 4C and S4C Fig), while *br* knockdown using a *br*^*RNAi*^ construct led to Chinmo maintenance during late larval stages (Fig 4D and S4D and S4E Fig). Additionally, *chinmo-lacZ* expression was aberrantly maintained when *br* is down-regulated in late L3 (S4F Fig), showing that Br also mediates the transcriptional repression of *chinmo*. We also tested other isoforms of Br; while misexpression of *br-Z2* is cell lethal, as shown by anti-Dcp-1 staining and pyknotic nuclei (S4G Fig), misexpression of *br-Z3* and *br-Z4* led to partial *chinmo* repression during early and mid L3 (S4H and S4I Fig). Together, these data demonstrate that Chinmo and Br form a bistable loop that ensures mutually exclusive expression during the development of the wing epithelium (Fig 4E).

### Chinmo maintains epithelial progenitors in an undifferentiated state

**Fig 5:**
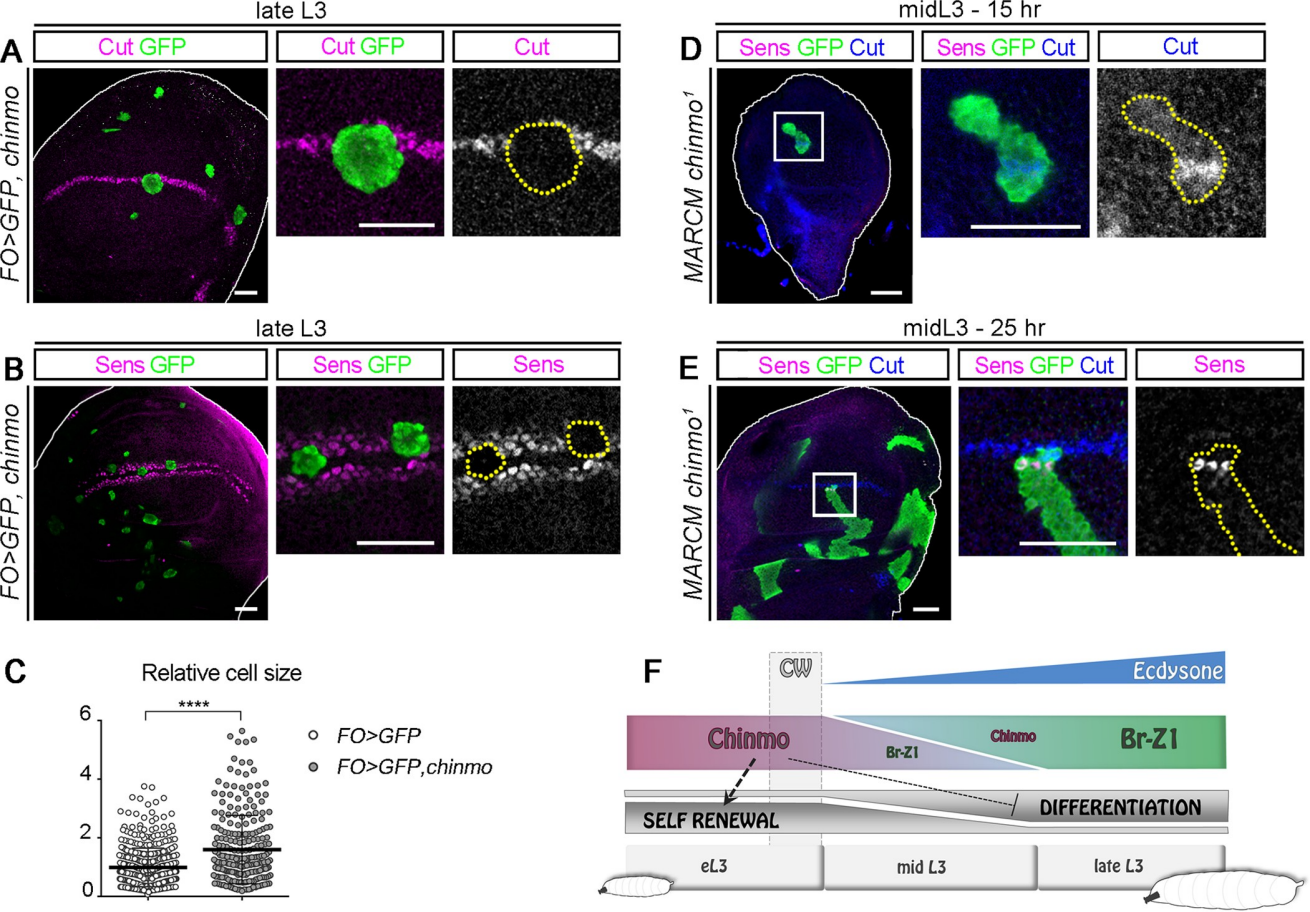
Chinmo prevents differentiation during L3 stages. *Flip-out* clones and MARCM clones are marked with GFP and outlined in yellow. (A–B) Anti-Cut (A, magenta) and anti-Sens (B, magenta) stainings are lost in late L3 *Flip-out* clones misexpressing *chinmo* (5/5 clones, *n* = 3 discs and 10/10 clones, *n* = 3 discs, respectively). (C) Relative cell area of wild-type and misexpressing *chinmo Flip-out* clone cells compared to wild-type surrounding cells in late L3. Wild-type *Flip-out* cells (*n* = 6 clones, 3 discs, 462 cells, m = 0.99 ± 0.03) and *chinmo* misexpressing cells (*n* = 9 clones, 3 discs, 245 cells, m = 1.60 ± 0.07). *p*-value is 3.5 × 10^−13^. (D–E) Anti-Cut (blue) and anti-Sens (magenta) stainings appear precociously in MARCM clones that are mutant for *chinmo* (5/5 clones, *n* = 5 discs and 3/3 clones, *n* = 3 discs, respectively). (F) Schematic outline of the above experiments. Scale bars: 30 **μ**m. *br*, *broad*; CW, critical weight; eL3, early L3; *FO*, *Flip-out*; GFP, green fluorescent protein; hr, hours; L3, third larval stage; MARCM, Mosaic Analysis with a Repressible Cell Marker *sens*, *senseless*.

We previously showed that in the neuroepithelium, Chinmo counteracts differentiation to promote self-renewal during early development [28]. We thus wondered if Chinmo was involved in counteracting differentiation of wing disc cells. Ecdysone production after the CW in mid L3 is required for activation of *sens* and *cut* proneural genes in cells that will form bristles along the wing margin [27,34,35,44,45] (S5A Fig). Since Chinmo down-regulation correlates with the activation of *cut* and *sens* (Fig 2A), we tested whether *chinmo* silencing was required to initiate the expression of these 2 genes. Misexpressing *chinmo* from L2 to late L3 led to a failure to activate *sens* and *cut* expression in the wing pouch (Fig 5A and 5B and S5B and S5C Fig). In addition, cells in clones misexpressing *chinmo* were larger than the surrounding Chinmo^-^ wild-type cells in late L3 (Fig 5C), consistent with a previous observation that wing cells are larger in earlyL3 than in late L3 [46]. Thus, *chinmo* misexpression in late larval stages is sufficient to prevent epithelial cell maturation and activation of the neurogenic differentiation program. In contrast, loss of Chinmo in MARCM *chinmo*^*1*^ mutant clones led to the premature expression of *cut* and *sens* in mid L3, although their activation remained sequential (Fig 5D and 5E). However, loss of *chinmo* during early L3, before the CW is reached, did not lead to *cut* expression (S5D Fig). This suggests that Chinmo down-regulation is not sufficient to activate proneural genes that require input from ecdysone signaling. Together, these data reveal that, during the early stages of imaginal disc development, Chinmo promotes a self-renewing state that is refractory to differentiation. Chinmo down-regulation after the CW is required for the timely initiation of differentiation programs induced upon ecdysone signaling in epithelial progenitors (Fig 5F).

### Br installs a differentiation-permissive state in wing disc epithelial progenitors

**Fig 6:**
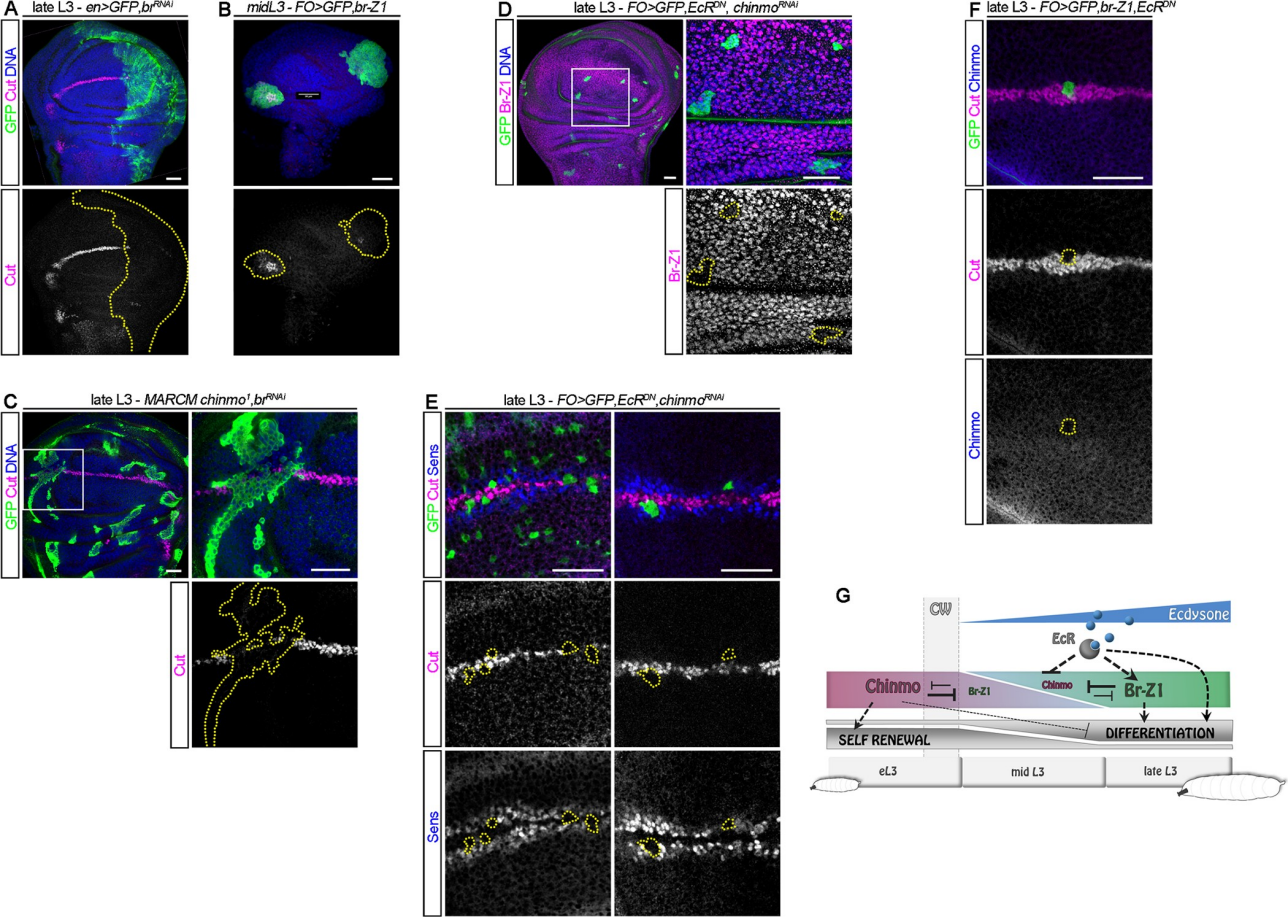
Ecdysone signaling and Br cooperate to induce differentiation. *Flip-out* clones, *GAL4* expression, and MARCM clones are marked with GFP and outlined in yellow. (A) Anti-Cut staining (magenta) is absent during late L3 when *br* is knocked down in the posterior compartment using *en-Gal4*. (B) *cut* (magenta) is ectopically expressed in *Flip-out* clone cells misexpressing *br-Z1* during mid L3. (C) *cut* (magenta) is not expressed in MARCM clones mutant for both *chinmo* and *br* (*chinmo*^*1*^;*br*^*RNAi*^ cells) during late L3 (11/12 clones, *n* = 7 discs). (D) *br-Z1* (magenta) is silenced in *Flip-out* clones expressing both *EcR*^*DN*^ and *chinmo*^*RNAi*^ transgenes in late L3 (52/52 clones, *n* = 4 discs). (E) *cut* (magenta) and *sens* (blue) are not expressed in *Flip-out* clones expressing both *EcR*^*DN*^ and *chinmo*^*RNAi*^ transgenes during late L3 (15/15 clones, *n* = 7 discs). (F) *cut* (magenta) is not expressed in *Flip-out* clones expressing both *br-Z1* and *EcR*^*DN*^ transgenes during late L3 (4/4, *n* = 3 discs). (G) Schematic outline of the above experiments. Scale bars: 30 **μ**m. *br*, *broad*; CW, critical weight; *EcR*^*DN*^, dominant negative form of ecdysone receptor; eL3, early L3; *en*, *engrailed*; *FO*, *Flip-out*; GFP, green fluorescent protein; L3, third larval stage; MARCM, Mosaic Analysis with a Repressible Cell Marker; RNAi, RNA interference; *sens*, *senseless*.

Recent studies have shown that, after the CW, ecdysone signaling and its downstream target *br* promote differentiation of wing disc epithelial cells [26,27,47]. Consequently, differentiation does not properly occur when *br* is knocked down in late L3 stages (Fig 6A and [26]). Conversely, clones of epithelial cells misexpressing *br-Z1* in mid L3 precociously expressed *cut* (Fig 6B). We wondered whether Br possesses both a permissive function by allowing Chinmo down-regulation and an instructive function by activating the expression of differentiation genes. For this purpose, we tested whether concomitant knockdown of *br* and *chinmo* restored differentiation. Loss of Br and Chinmo in *chinmo*^*1*^*;br*^*RNAi*^ MARCM clones failed to activate differentiation genes in late L3 (Fig 6C). Thus, Br is indeed instructive: it is required to activate a differentiation program that is not a default state when *chinmo* is silenced. We thereafter tested whether *br* activation required input from ecdysone signaling in addition to *chinmo* down-regulation. Interestingly, Br-Z1 levels are markedly reduced in *EcR*^*DN*^*;chinmo*^*RNAi*^ *Flip-out* clones (Fig 6D). Thus, *chinmo* silencing is not sufficient for the optimal activation of *br* that requires input from ecdysone signaling. In addition, we show that wing epithelial cells that coexpress both *EcR*^*DN*^ and either *chinmo*^*RNAi*^ or *br-Z1* fail to properly differentiate (Fig 6E and 6F). Thus, while Chinmo represses differentiation and Br favors it, activation of differentiation programs requires other inputs from ecdysone signaling. Altogether, our data demonstrate that ecdysone signaling after the CW induces a switch in epithelial wing disc cells: from a default proliferative and differentiation-refractory state defined by Chinmo to a differentiation-permissive state defined by Br (Fig 6G).

### The ecdysone-mediated Chinmo-to-Br switch restricts the regenerative potential of epithelia

**Fig 7:**
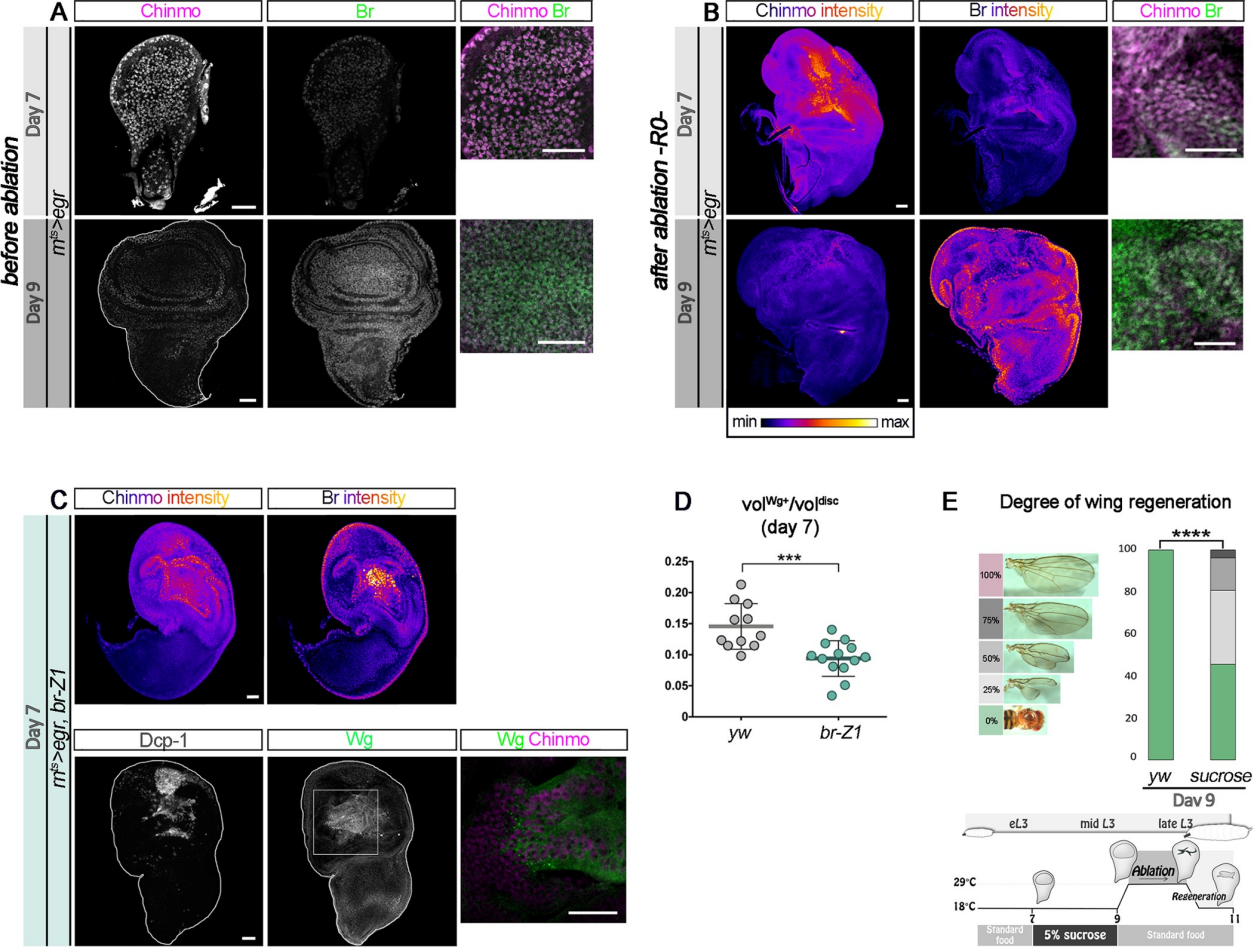
The Chinmo-to-Br switch restricts regenerative capacity. (A) Before d7 ablation, Chinmo (magenta) is highly expressed and Br (green) is low, whereas Chinmo is low and Br is highly expressed at d9. (B) Anti-Chinmo and anti-Br stainings (color-coded relative to staining intensity) at R0 in a *rn*^*ts*^*>egr* wing disc after d7 and after d9 ablations. Blow-up shows that Chinmo (magenta) is high in the wing pouch while Br is low (green) at d7. In contrast, Chinmo is low in the wing pouch while Br is strong at d9. (C) Anti-Chinmo and anti-Br stainings (both color-coded relative to staining intensity) and anti-Dcp-1 (gray), anti-Wg (green), and anti-Chinmo (magenta in the blow-up) stainings in an *rn*^*ts*^*>egr*,*br-Z1* wing disc at R0 after d7 ablation. Chinmo and Wg are low while Br is high in the wing pouch. (D) Volume of anti-Wg staining over total wing disc volume at R0 upon d7 ablation in *rn*^*ts*^*>egr* larvae (*n* = 11 wing discs, m = 0.145 ± 0.011) and *rn*^*ts*^*>egr*,*br-Z1* larvae (*n* = 13 wing discs, m = 0.094 ± 0.008). *p* = 9.2 × 10^−4^. (E) Distribution of degrees of wing regeneration upon d9 ablation in *rn*^*ts*^*>egr* adults (*n* = 122 wings) and *rn*^*ts*^*>egr* adults grown on sucrose 5% from d7 to d9 (*n* = 162 wings). *p* = 5.1 × 10^−21^. Scheme depicting the timing of starvation and ablation procedure. Scale bars: 30 **μ**m. Underlying data for Fig 7 can be found in S1 Data. *br*, *broad*; d, day; Dcp-1, Death Caspase-1; *egr*, *eiger*; eL3, early L3; L3, third larval stage; *rn*^*ts*^, *rotund-GAL4*, *tubulin-GAL80*^*thermo-sensitive*^; R0, beginning of the recovery period; vol, volume; Wg, Wingless; *yw*, *yellow*,*white*.

Early ectopic exposure to ecdysone restricts the window of regenerative competence of wing epithelial progenitors [18]. However, the underlying mechanisms remain unclear. In particular, it is unknown whether ecdysone functions cell-autonomously or non-cell-autonomously. Given the respective function of Chinmo and Br in promoting self-renewal and differentiation during development and the necessity of *chinmo* expression for efficient regeneration, we tested whether the Chinmo-to-Br switch by ecdysone could be responsible for restricting the regenerative potential of imaginal disc epithelia during the late L3 stage. First, we assessed the state of *chinmo* and *br* expression at d7, when discs are regeneration competent, and at d9, when discs are regeneration incompetent. We observed high Chinmo and low Br levels in wing discs at d7, which corresponds to late L2/early L3 larvae (Fig 7A). In contrast, Chinmo was low or absent while Br was high at d9, which therefore corresponds to mid L3 (between 20 to 30 hours on Fig 2D, Fig 7A). Thus, Chinmo and Br levels at the time of ablation are indicative of the regenerative potential of wing imaginal discs. Moreover, these stainings show that larvae at d9 have passed the CW. Additionally, Chinmo is still highly expressed throughout the disc at the end of the ablation window (R0), with mild levels of Br, when damages are induced at d7, whereas Chinmo levels outside the blastema remain low or null and Br levels are high when damages are induced at d9 (Fig 7B). This is consistent with ablation causing a block in developmental progression when induced in early L3 (d7).

Knowing that Chinmo facilitates efficient regeneration (Fig 1), we tested whether Br could counteract efficient regeneration. Consistent with this hypothesis, we found that misexpression of *br-Z1* in d7-damaged discs reduced Chinmo levels in the blastema and the ratio of Wg-expressing cells at R0 compared to control (Fig 7C and 7D and S6A and S6B Fig), indicating a restricted regenerative response. Misexpression of *br-Z1* using the *rn*^*ts*^*>egr* system caused lethality at early pupal stages, and we were not able to assess regeneration in adults. Nevertheless, these data strongly suggest that Chinmo and Br antagonistically regulate the competency to activate a regenerative response. Consistent with this hypothesis, blocking developmental progression by transferring early L3 (d7) larvae on a sucrose-only diet until d9, then performing the ablation protocol back on the normal food, leads to a third of adults depicting 25% or more wing regeneration, compared to an absence of regeneration in control d9 flies (Fig 7E). Thus, stalling developmental progression before the CW can extend the time window of regenerative competence. Together, these results suggest that the Chinmo-to-Br switch triggered by reaching the CW restricts the regenerative potential of wing imaginal disc cells.

### Manipulations of the ecdysone pathway can enhance the regenerative potential of late larval discs

**Fig 8:**
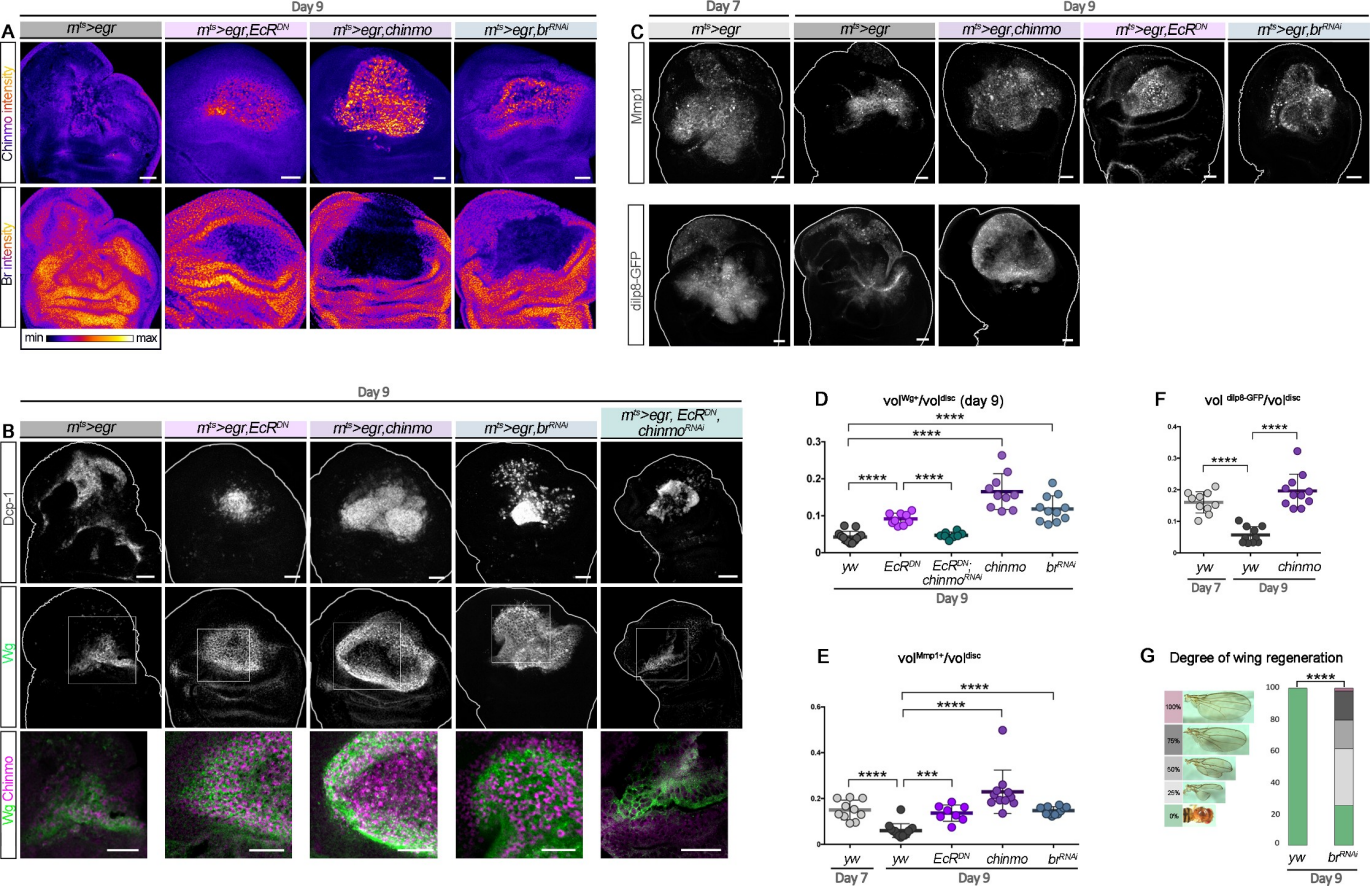
Preventing the Chinmo-to-Br switch can restore regenerative potential in late L3. (A) Anti-Chinmo and anti-Br wing disc stainings at R0 after d9 ablation in various genetic conditions (color-coded relative to staining intensity). (B) Anti-Dcp-1 (gray) and anti-Wg (green) wing disc stainings at R0 after d9 ablation in various genetic conditions. Blow-up shows that *wg* and *chinmo* (magenta) are highly coexpressed in the blastema of *rn*^*ts*^*>egr*,*EcR*^*DN*^; *rn*^*ts*^*>egr*,*chinmo*; and *rn*^*ts*^*>egr*,*br*^*RNAi*^ discs, whereas Chinmo and Wg are lower in the blastema of *rn*^*ts*^*>egr* and *rn*^*ts*^*>egr*,*EcR*^*DN*^,*chinmo*^*RNAi*^ discs. (C) Anti-Mmp1 (gray) staining at R0 after d7 and d9 ablation in various genetic conditions. Dilp8-GFP (gray) is poorly expressed in d9 *rn*^*ts*^*>egr yw* wing discs, whereas it is highly expressed in d7 *rn*^*ts*^*>egr*,*yw* and d9 *rn*^*ts*^*>egr*,*chinmo* wing discs. (D) Volume of anti-Wg staining over total wing disc volume at R0 upon d9 ablation at in *rn*^*ts*^*>egr* larvae (*n* = 13 wing discs, m = 0.043 ± 0.004); *rn*^*ts*^*>egr*,*EcR*^*DN*^ larvae (*n* = 9 wing discs, m = 0.092 ± 0.005); *rn*^*ts*^*>egr*,*EcR*^*DN*^,*chinmo*^*RNAi*^ larvae (*n* = 8 wing discs, m = 0.047 ± 0.003); *rn*^*ts*^*>egr*,*chinmo* larvae (*n* = 10 wing discs, m = 0.165 ± 0.015); and *rn*^*ts*^*>egr*,*br*^*RNAi*^ larvae (*n* = 11 wing discs, m = 0.089 ± 0.011). *p* = 8.0 × 10^−6^, *p* = 8.2 × 10^−5^, *p* = 1.7 × 10^−6^, and *p* = 8.0 × 10^−7^ (*rn*^*ts*^*>egr* compared to *rn*^*ts*^*>egr*,*EcR*^*DN*^; *rn*^*ts*^*>egr*,*EcR*^*DN*^ compared to *rn*^*ts*^*>egr*,*EcR*^*DN*^,*chinmo*^*RNAi*^; *rn*^*ts*^*>egr* compared to *rn*^*ts*^*>egr*,*chinmo*; and *rn*^*ts*^*>egr* compared to *rn*^*ts*^*>egr*,*br*^*RNAi*^, respectively). (E) Volume of anti-Mmp1 staining over total wing disc volume at R0 upon d7 ablation in *rn*^*ts*^*>egr* larvae (*n* = 10 wing discs, m = 0.150 ± 0.014) and upon d9 ablation in *rn*^*ts*^*>egr* larvae (*n* = 14 wing discs, m = 0.060 ± 0.008), *rn*^*ts*^*>egr*,*chinmo* larvae (*n* = 11 wing discs, m = 0.230 ± 0.029), *rn*^*ts*^*>egr*,*EcR*^*DN*^ larvae (*n* = 8 wing discs, m = 0.136 ± 0.013), and *rn*^*ts*^*>egr*,*br*^*RNAi*^ larvae (*n* = 12 wing discs, m = 0.147 ± 0.005). *p* = 1.9 × 10^−5^, *p* = 89.0 × 10^−7^, *p* = 1.9 × 10^−4^, and *p* = 6.2 × 10^−6^ (d7 *rn*^*ts*^*>egr* compared to d9 *rn*^*ts*^*>egr* and d9 *rn*^*ts*^*>egr* compared to *rn*^*ts*^*>egr*,*chinmo*, to *rn*^*ts*^*>egr*,*EcR*^*DN*^, and to *rn*^*ts*^*>egr*,*br*^*RNAi*^, respectively). (F) Volume of anti-dilp8-GFP staining over total wing disc volume at R0 upon d7 ablation in *rn*^*ts*^*>egr* larvae (*n* = 10 wing discs, m = 0.160 ± 0.011) and upon d9 ablation in *rn*^*ts*^*>egr* larvae (*n* = 10 wing discs, m = 0.057 ± 0.008) and in *rn*^*ts*^*>egr*,*chinmo* larvae (*n* = 11 wing discs, m = 0.200 ± 0.016). *p* = 2.2 × 10^−5^ and *p* = 5.7 × 10^−6^ (d7 *rn*^*ts*^*>egr* compared to d9 *rn*^*ts*^*>egr* and d9 *rn*^*ts*^*>egr* compared to d9 *rn*^*ts*^*>egr*,*chinmo*, respectively). (G) Distribution of degrees of wing regeneration in *rn*^*ts*^*>egr*,*yw* adults (*n* = 216 wings) and *rn*^*ts*^*>egr*,*br*^*RNAi*^ adults (*n* = 172 wings) after d9 ablation. *p* = 4.4 × 10^−33^. Scale bars: 30 **μ**m. Underlying data for Fig 8 can be found in S1 Data. *br*, *broad*; d, day; Dcp-1, Death Caspase-1; Dilp8, *Drosophila* Insulin-like peptide 8; *EcR*^*DN*^, dominant negative form of ecdysone receptor; *egr*, *eiger*; GFP, green fluorescent protein; L3, third larval stage; Mmp1, Matrix metalloprotease 1; RNAi, RNA interference; *rn*^*ts*^, *rotund-GAL4*, *tubulin-GAL80*^*thermo-sensitive*^; R0, beginning of the recovery period; vol, volume; Wg, Wingless; *yw*, *yellow*,*white*.

We then tested whether transiently re-expressing *chinmo* in wing pouch cells in late larvae could enhance the ability to regenerate upon late ablation (d9). Given the regulatory relationships between *chinmo*, *br*, and ecdysone signaling, three types of genetic interventions were performed: transient misexpression of *chinmo*, transient silencing of ecdysone signaling, and transient silencing of *br*. For this purpose, transient misexpression of *chinmo*, *EcR*^*DN*^, and *br*^*RNAi*^ using the *rn*^*ts*^*>egr* system all led to high levels of Chinmo in the regenerating blastema at R0 (Fig 8A). We used POU domain Protein/Nubbin (Pdm1/Nub) to label wing pouch cells. Strikingly, in all conditions, the Pdm1/Nub^+^ blastema exhibited a much less folded appearance compared to control, possibly indicating a more efficiently regenerated wing pouch (S7A Fig). Consistently, all conditions were associated with high levels of regeneration markers such as Wg, the Matrix metalloprotease 1 (Mmp1), and the relaxin-like peptide *Drosophila* Insulin-like peptide 8 (Dilp8) [13,48–50] throughout the large blastema (*dilp8* expression was only tested in the *chinmo* misexpression condition) (Fig 8B–8F and S7B and S7C Fig). Note that for an unknown reason, misexpression of *chinmo* was associated with a strong down-regulation of *pdm1/nub* in the center of the blastema (S7A Fig). Interestingly, misexpression of *EcR*^*DN*^ in the pouch of undamaged imaginal discs during late L3, although leading to ectopic Chinmo, did not trigger ectopic Wg (S7D Fig). Thus, misexpression of *EcR*^*DN*^ only leads to strong ectopic *wg* in the context of tissue damage, reflecting activation of a regenerative response. Intriguingly, upon regeneration, apoptosis appeared much less pronounced in the *EcR*^*DN*^ condition than in all other tested conditions (S7E Fig), suggesting that the maintenance of a “younger” early L3-like state may protect against *egr*-mediated apoptosis. A similar antiapoptotic effect has recently been described upon early d7 ablation and shown to be mediated by high levels of JAK/STAT signaling [49]. Interestingly, knockdown of *chinmo* in *EcR*^*DN*^ cells of the wing pouch (*UAS-EcR*^*DN*^, *UAS-chinmo*^*RNAi*^) did not restore high apoptosis but reduced the size of the Wg^+^ blastema compared to the *EcR*^*DN*^ condition (Fig 8D and S7E Fig). Moreover, the *br*^*RNAi*^ and *chinmo* misexpression conditions also exhibited high levels of apoptosis (S7E Fig). Thus, inhibition of apoptosis in the *EcR*^*DN*^ condition is not mediated by Chinmo. In contrast, the decreased Wg volume in *EcR*^*DN*^, *chinmo*^*RNAi*^ suggests that the enhanced regenerative response induced by *EcR*^*DN*^ upon late ablation at least partly relies on Chinmo (Fig 8B–8D and S7B and S7C Fig). We then sought to assess the efficiency of the regenerative process by looking at adult wings. Ectopic expression of *EcR*^*DN*^ or *chinmo* using the *rn*^*ts*^*>egr* system led to lethality during pupariation, leaving us unable to assess the extent of regeneration in adult flies. However, the system led to viable flies in the *br*^*RNAi*^ condition. While wings failed to regenerate upon *egr*-mediated ablation at d9, as assessed by the systematic absence of wings in adults (Fig 8G), more than 70% of the adult flies in which *br* was transiently knocked down exhibited partial to almost complete regeneration (Fig 8G). Similar results were obtained using the *rn*^*ts*^*>rpr* system (S1E Fig). Thus, transient down-regulation of Br can revert late wing imaginal disc cells into an earlier Chinmo^+^ regeneration-competent state. Together, these results strongly suggest that ecdysone signaling restricts regenerative abilities in late larvae by increasing sensitivity to *egr*-mediated apoptosis and by decreasing the regenerative response via the Chinmo-to-Br switch.

## Discussion

**Fig 9:**
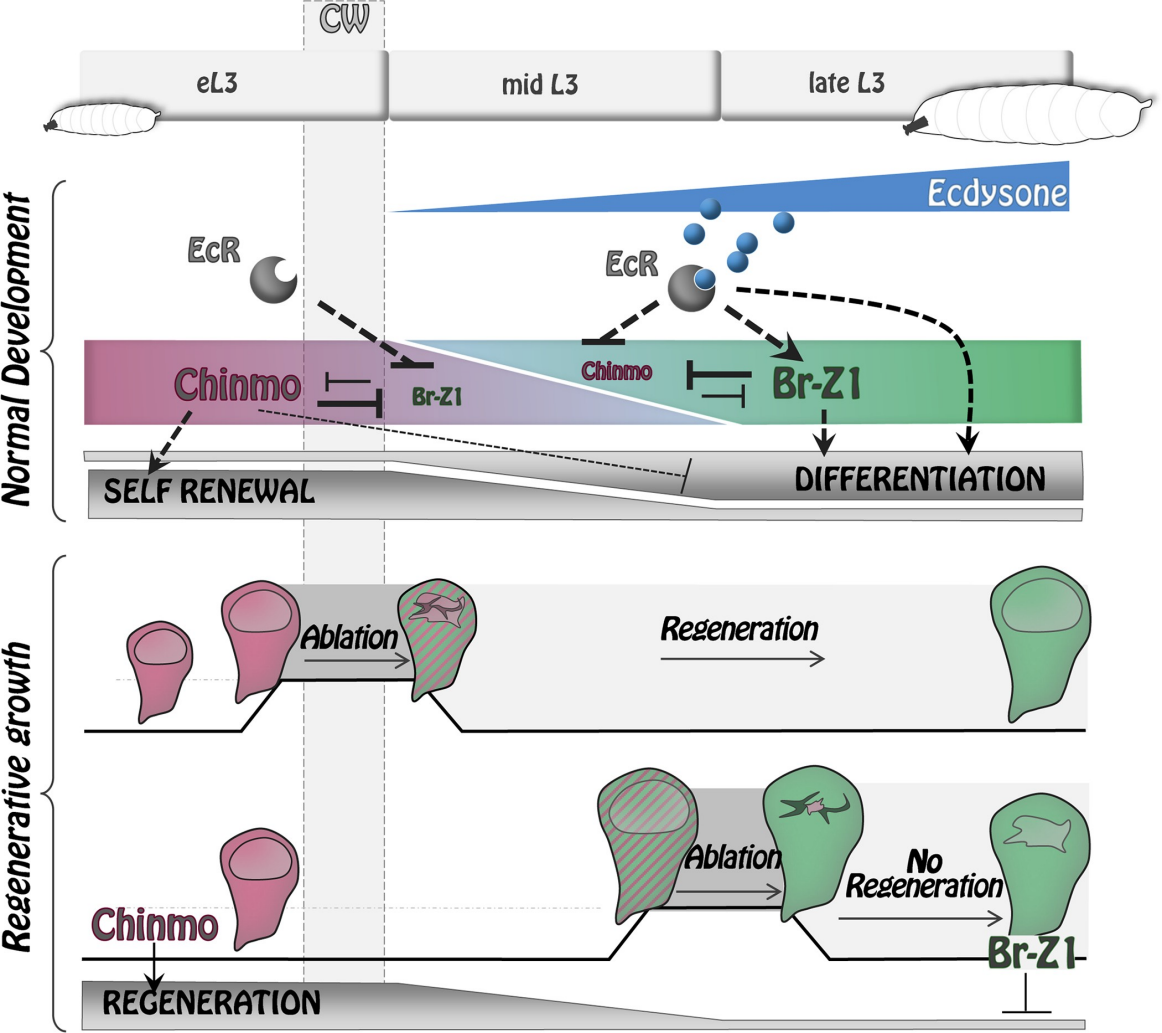
Ecdysone coordinates self-renewal, differentiation, and regenerative potential with developmental progression via the Chinmo/Br bistable loop. During early development, Chinmo represses differentiation programs while promoting self-renewal state in wing disc epithelial cells. Upon ecdysone production after the CW, *br* becomes activated and promotes differentiation by repressing Chinmo and possibly other genes. The Chinmo-to-Br switch induced by ecdysone also causes restriction of regenerative potential. *br*, *broad*; CW, critical weight; *EcR*, ecdysone receptor; eL3, early L3; L3, third larval stage.

Here, we identify a bistable switch downstream to ecdysone that modifies the differentiation and regenerative properties of wing imaginal disc cells during larval development in *Drosophila*. We demonstrate that this change in cell properties can essentially be attributed to the sequential expression of two mutually exclusive ZBTB transcription factors, Chinmo and Br, that form a bistable loop through cross-repression. It has been formerly proposed that *chinmo* is a positive target of JAK/STAT signaling in early eye discs and other tissues [29]. However, JAK/STAT signaling in wing imaginal discs exhibits a different spatiotemporal regulation than *chinmo*. In particular, JAK/STAT signaling remains strong in the hinge region of wing discs in late L3 [51–53], while *chinmo* is silenced throughout the disc. Therefore, *chinmo* does not seem to be regulated by JAK/STAT signaling in the wing disc. Instead, we have found that before the CW, Chinmo is uniformly high throughout wing imaginal discs and appears to propagate a default self-renewing state that is refractory to differentiation. When the CW is reached (8–12 hours after the L2/L3 molt), ecdysone is produced by the prothoracic gland, and its mature form, 20-HE, binds to its nuclear receptor expressed by imaginal disc cells. This event triggers *br* expression, which then leads to complete repression of *chinmo* throughout the disc by 30 hours. Our results indicate that *Br-Z1* is likely to be the major isoform involved in *chinmo* repression because other isoforms are either not expressed or less efficient in repressing *chinmo*. *chinmo* progressive repression after the CW turns imaginal tissues into a differentiation-permissive state. After *chinmo* silencing, ecdysone signaling and *br* expression cooperate to activate differentiation cascades leading to wing disc cells progressively acquiring their terminal fate—as observed for neural specification at the dorsoventral boundary. Our work therefore provides a mechanism by which ecdysone signaling coordinates the end of a default self-renewing state and the initiation of terminal differentiation programs in imaginal discs with organismal growth (Fig 9).

In principle, when larvae are starved before the CW, this mechanism also permits locking cells into a Chinmo^+^ differentiation-refractory state, therefore ensuring that differentiation cascades in imaginal discs remain repressed as organismal growth is stopped. Thus, linking the Chinmo-to-Br switch to the CW allows the stalling of developmental progression during starvation periods that may occur during early larval development and protects the developmental potential of early epithelial progenitors until nutrients become abundant again.

Recently, a study has identified a sequence of ecdysone-induced transcription factors responsible for the progressive maturation of wing imaginal disc cells during the late L3 to pupal transition [54]. Interestingly, Br was positioned first in this cascade. Although it is still unclear how cross-regulatory interactions between ecdysone-induced transcription factors drives progression throughout the sequence, we propose that a first step in this cascade is the silencing of Chinmo through the cooperative action of ecdysone signaling and Br after the CW.

Our lab has recently shown that Chinmo also sustains self-renewal and prevents differentiation in other types of progenitors during early development, such as in the early neuroepithelium of optic lobes and in early NBs of the ventral nerve cord and central brain [28,30,38]. Moreover, aberrant expression of *chinmo* propagates the growth of NB tumors induced by the inactivation of *prospero* or *brat* [38] and of eye disc tumors induced by the inactivation of the Polycomb-Group (PcG) gene *polyhomeotic* [55]. Thus, aberrant maintenance of a default Chinmo^+^ state beyond early development is emerging as a widespread tumor-propagating mechanism in *Drosophila* tumors with early developmental origins.

The temporal regulation of *chinmo* and *br* during wing imaginal disc development seems to be largely responsible for the decrease of regenerative potential observed during progression throughout the L3 stage. We find that *chinmo* is highly expressed in the regenerating blastema upon wing pouch ablation performed before the CW and is required for efficient regeneration. In contrast, *chinmo* is less efficiently expressed when ablation is performed after the CW (d9), and *chinmo* misexpression in this context is sufficient to trigger high expression of regeneration markers such as Wg, Mmp1, and Dilp8. Transient misexpression of *EcR*^*DN*^ or *br*^*RNAi*^ upon late ablation (d9) also leads to potent activation of *chinmo* and regeneration markers. In addition, both genetic interventions appear to enhance regeneration upon late ablation, as assessed by the enlarged blastema size at R0 and the efficient regenerative process observed in adult wings (only tested for the *br*^*RNAi*^ condition). Together, these data suggest that ecdysone production after the CW triggers a switch from a regeneration-permissive to a regeneration-refractory state that is, at least partly, mediated by the Chinmo-to-Br transition. This mechanism provides a molecular link between developmental progression and progressive restriction of regenerative potential (Fig 9).

Interestingly, *wg* is not ectopically induced when *EcR*^*DN*^ is misexpressed in an undamaged late L3 wing pouch, despite ectopic expression of *chinmo*. Thus, Chinmo is not sufficient to trigger *wg* expression but rather seems to establish/maintain the competence for imaginal disc cells to respond to signals induced upon damage (such as JNK) by activating widespread expression of *wg* and other pro-regeneration genes. It has recently been shown that imaginal discs are subjected to a partial remodeling of their PcG-mediated chromatin landscape between early and late L3 [56]. This redistribution of a subset of PcG-binding sites may underlie the progressive restriction of regenerative potential. In particular, PcG-mediated repression at a specific *wg* enhancer prevents efficient activation upon damage in late L3 [13]. Our study implies that Chinmo may be able to overcome or prevent the establishment of PcG-mediated repression at the *wg* enhancer. Interestingly, unlike for *chinmo*, the misexpression of *wg* alone is not sufficient to restore regenerative potential [13]. Thus, Chinmo may be able to alleviate the PcG-mediated repression established at other genes in late L3, favoring transient reprogramming to an earlier differentiation-refractory state that is regeneration competent. On the other hand, Br can restrict regeneration potential via the repression of *chinmo* and possibly other genes. It remains to be shown whether Br contributes to the redistribution of PcG binding during L3. Altogether, our work raises the exciting possibility of Chinmo and Br acting as two antagonistic pioneer factors [57] that link developmental progression to changes in chromatin landscapes to establish various competence states.

Chinmo and Br both belong to the family of ZBTB transcription factors [32,58,59]. In mammals, ZBTB transcription factors are involved in a large array of functions during development and malignancy (reviewed in [60–62]). They are usually associated with a repressive activity and appear to work in conjunction with chromatin factors, although their mode of action is still poorly understood [62]. Unravelling how the antagonistic activities of Chinmo and Br may translate at the chromatin and transcriptional levels offers the opportunity to reveal fundamental principles that could allow transient cell rejuvenation for improved regenerative therapies.

## Materials and methods

### Fly culture

*Drosophila* stocks were maintained at 18°C on standard medium (8% cornmeal, 8% yeast, 1% agar). To assess the effects of rearing larvae on sucrose-only medium, larvae that ecdysed from L2 to L3 were transferred to a medium of 1% agar, 5% sucrose in water for 48 hours.

### Image processing

Confocal images were acquired on a Zeiss LSM780 microscope (Zeiss, Oberkochen, Germany). FIJI was used to process confocal data. In each picture, the scale bar represents 30 **μ**m.

To measure the “Wg, Mmp1, or dilp8-GFP volumes/wing disc volume ratio” upon ablation, Z-stacks through wing discs (Z-step of 3.5 **μ**m) stained with an anti-Wg, anti-Mmp1, or anti-GFP and 4′,6-diamidino-2-phenylindole (DAPI) were taken. For each image, masks were manually obtained by applying a Gaussian blur (sigma radius = 2) and then the Threshold function (settings: isoData, dark background) in FIJI [63]. For each stack, volumes were reconstituted and quantified using the “3D object counter analyser” in FIJI. For Wg measurements only, the Wg signal outside the wing pouch was manually removed before applying the Gaussian blur.

To measure relative Wg intensity, for a given focal plane, the mean intensity of Wg staining in Wg-positive cells was divided by the mean intensity of Wg staining in Wg-negative cells located outside of the wing pouch delineated by morphological criteria revealed by DAPI. Both intensities were obtained with the “Measure” analyzer in FIJI. This ratio has been calculated for the three focal planes showing highest Wg intensity in the disc. Each dot on the graphs represents the mean of these three ratios.

To measure relative Chinmo intensity for regeneration experiments, the same procedure as for Wg was followed.

To measure relative Chinmo and Br intensities in *EcR*^*RNAi*^ *Flip-out* clones, for each clone, one single confocal image corresponding to the focal plane where nuclei are localized was acquired. The mean intensity of Chinmo (or Br) staining throughout a clone was obtained with the Measure analyzer in FIJI and divided by the intensity of Chinmo (or Br) staining in a surrounding area of similar size outside of the clone in the same focal plan. One dot corresponds to the ratio between these 2 mean intensities. The subsequent relative intensities are plotted on a log_10_ scale.

To measure cell size, the area of each cell within a wild-type or *chinmo Flip-out* clone was measured by applying the Smooth and Sharpen process functions on the DAPI mask, then the Find Maxima function (noise tolerance: 30, output type: segmented particles, exclude edge maxima) in FIJI. Clones (marked with the GFP) and wild-type surrounding cells (at least 79 cells) were manually delimitated on the segmented image and measured using the Analyze particles function (size: 1–infinity, exclude on edge). The relative size for each clone cell was the ratio between the area of the cell and the mean area of the wild-type surrounding cells.

### Statistical analysis

For all the experiments except the range of adult wing size experiments, we performed a Mann–Whitney test for statistical analysis. No data were excluded. Statistical tests and graphs were performed with Prism. Results are presented as dot plots, also depicting the median and the Standard Deviation (Whisker mode: 1.5IQR). The sample size (*n*), the mean ± the standard error of the mean (m ± SEM), and the *p*-value are reported in the figure legends. For the range of adult wing size experiments, we performed an exact Fisher test. The sample size (*n*) and the *p*-values are reported in the figure legends.

****p-value ≤ 0.0001, ***p-value ≤ 0.001, **p-value ≤ 0.01, and *p-value ≤ 0.05.

### Fly lines

Experiments were performed at 25°C or 29°C except for the regeneration experiments. Crosses to *yellow*,*white* (*yw*) line are used as controls. For generating *chinmo*^*1*^ MARCM clones [64], we used *yw*,*hs-FLP;FRT40A*,*tubulin-GAL80/CyO*,*actin-GFP;tubulin-GAL4*,*UAS-mCD8-GFP/TM6* (from P. Speder) crossed to *chinmo*^*1*^,*UAS-mCD8-GFP*,*FRT40A/CyO* [32] or *chinmo*^*1*^,*UAS-mCD8-GFP*,*FRT40A/CyO;UAS-br*^*RNAi*^*/TM6* (from Transgenic RNAi Project [TRiP] #HMS00042, Bloomington #33641; Bloomington *Drosophila* Stock Center, Bloomington, IN, USA). *Flip-out* clones were generated using *hs-FLP;Actin5c>CD2>GAL4*,*UAS-GFP* (from N. Tapon) or *hs-FLP;Actin5c>CD2>GAL4*,*UAS-RFP/TM6* (from Bloomington #7 and #30558). The progeny of the crosses were heat-shocked 1 hour at 37°C just after larval hatching. The GAL4 lines used were the following: *nab-GAL4* (#6190 from Kyoto *Drosophila* Genetic Resource Consortium (DGRC), [65] and *en-GAL4* (Bloomington #30564). The UAS lines used were *UAS-chinmo*^*FL*^ (Bloomington #50740), *UAS-HA-chinmo* [29], *UAS-br*^*RNAi*^*/TM6* (from TRiP #HMS00042, Bloomington #33641), *UAS-br-Z1* (Bloomington #51379), *UAS-EcRcore*^*RNAi*^ [66], *UAS-EcR*^*DN*^ (*UAS-EcR*.*B1*.*W650A*, Bloomington #6872 or *UAS-EcR*.*A*.*W650A*, Bloomington #9451), and *UAS-chinmo*^*RNAi*^*/TM6* (from TRiP #HMS00036, Bloomington #33638). *UAS-dicer2* (Bloomington #24650 and #24651) was used in combination with GAL4 lines in order to improve RNAi efficiency. *UAS-p35* (Bloomington #5072) was used to inhibit apoptosis. *UAS-mCD8*::*GFP* (Bloomington #32186) was used to follow the GAL4 driver activity. The *chinmo-lacZ* line (Bloomington #10440) was used to monitor *chinmo* transcription, and the *UAS-mCherry*^*chinmoUTR*s^ line was used to follow *chinmo* post-transcriptional regulation [28]. The *dilp8-GFP* line (Bloomington #33079) was used to follow *dilp8* expression.

For each experiment using the *GAL4* system, more than 30 discs have been observed.

For ablation experiments, we let the flies lay for 24 hours at 18°C. Progeny were maintained at 18°C, switched to 29°C for 40 hours after 7 or 9 days, and put back at 18°C until adult hatching The “ablation” line, *w*^*1118*^*;rotund-GAL4*,*tubulin-GAL80*^*ts*^,*UAS-egr/TM6B*,*tubulin-GAL80* (*rn*^*ts*^*>egr*, from I. K. Hariharan), was crossed to *yw*, *UAS-chinmo*^*RNAi*^*/TM6*, *UAS-br-Z1*, *UAS-br*^*RNAi*^*/TM6*, *UAS-chinmo*^*FL*^, or *UAS-HA-chinmo* and *UAS-EcR*^*DN*^.

The larval stages are standardized using morphological criteria. L2 and L3 larval stages were discriminated based on anterior spiracle morphology. EarlyL3 larvae may have a similar size as late L2 larvae but exhibit “open” anterior spiracles.

### Immunohistochemistry

Dissected tissues were fixed 5 to 20 minutes in 4% formaldehyde/PBS depending on the primary antibody. Stainings were performed in 0.5% Triton/PBS with antibody incubations separated by several washes. Tissues were then transferred in Vectashield with or without DAPI for image acquisition. Primary antibodies were chicken anti-GFP (1:1,000, Aves #GFP-1020), rabbit anti-RFP (1:500, Rockland #600-401-379), rat anti-RFP (1:500, Chromotek #5F8), mouse anti-Cut (1:50, Developmental Studies Hybridoma Bank [DSHB] #2B10), mouse anti-Br-core (1:50, DSHB #25E9.D7), mouse anti-Br-Z1 (1:50, DSHB #Z1.3C11.OA1), mouse anti-Br-Z3 (1:50, DSHB #Z3.9A7), rabbit anti-Br-Z2 (1:50, Y. Song), mouse anti-Mmp1 (1:100, a combination of DSHB #14A3D2, 3A6B4, and 5H7B11), mouse anti-EcR (1:7, DSHB #Ag10.2), rabbit anti-ß-galactosidase (1:1,000, Cappel #559562), guinea pig anti-Sens (1:1,000, H. Bellen), mouse anti-Wg (1:100, DSHB #4D4), rabbit anti-Pdm1/Nub (1:500, S. Cohen), rat anti-Chinmo (1:500, N. Sokol), and guinea pig anti-Chinmo (1:500, N. Sokol). Rabbit anti-cleaved Dcp-1 (1:500, Cell Signaling #9578) labels apoptotic cells. Adequate combinations of secondary antibodies (Jackson ImmunoResearch) were used to reveal expression patterns.

## Supporting information

S1 Fig Wing regeneration with the *rn*^*ts*^*>rpr* system. (A) Anti-Wg (green), anti-Chinmo (magenta), and anti-Dcp-1 (blue) stainings in an undamaged late L3 wing disc. (B) Relative anti-Wg staining intensity in the wing pouch of *rn*^*ts*^*>egr* larvae at R0 after d7 ablation (*n* = 13 wing discs, m = 2.57 ± 0.13), of *rn*^*ts*^*>egr* at R0 after d9 ablation (*n* = 10 wing discs, m = 2.01 ± 0.12), and of *rn*^*ts*^*>egr*,*chinmo*^*RNAi*^ larvae at R0 after d7 ablation (*n* = 10 wing discs, m = 1.88 ± 0.10). *p* = 0.0080 and *p* = 0.0005 (*rn*^*ts*^*>egr*,*yw* at d7 compared to *rn*^*ts*^*>egr*,*yw* at d9 and *rn*^*ts*^*>egr*,*yw* at d7 compared to *rn*^*ts*^*>egr*,*chinmo*^*RNAi*^ at d7, respectively). (C) Relative anti-Chinmo staining intensity in the wing pouch of *rn*^*ts*^*>egr* larvae at R0 after d7 ablation (*n* = 13 wing discs, m = 2.01 ± 0.09), of *rn*^*ts*^*>egr* larvae at R0 after d9 ablation (*n* = 12 wing discs, m = 1.65 ± 0.06), and of *rn*^*ts*^*>egr*,*chinmo*^*RNAi*^ larvae at R0 after d7 ablation (*n* = 11 wing discs, m = 1.61 ± 0.18). *p* = 0.0055 and *p* = 0.0025 (*rn*^*ts*^*>egr*,*yw* at d7 compared to *rn*^*ts*^*>egr*,*yw* at d9 and *rn*^*ts*^*>egr*,*yw* at d7 compared to *rn*^*ts*^*>egr*,*chinmo*^*RNAi*^ at d7, respectively). (D) Chinmo (magenta) is low in dying cells outlined in yellow, marked by Dcp-1 staining (green) and pyknotic nuclei seen with DAPI staining (blue). (E) Schematic representation of the *rn*^*ts*^*>rpr* ablation system used to induce wing pouch ablation. Strong *wg* expression at R0 is observed in response to damage when ablation is initiated at d7 for 20 hours. *wg* expression is drastically reduced when ablation is initiated at d9. From [9]. (F) Examples of wing size scores are shown. Distribution of wing size from *rn*^*ts*^*>rpr*,*yw* adults after d7 ablation (*n* = 1,217 wings); *rn*^*ts*^*>rpr*,*chinmo*^*RNAi*^ adults after d7 ablation (*n* = 186 wings); *rn*^*ts*^*>rpr*,*yw* adults after d9 ablation (*n* = 185 wings); and *rn*^*ts*^*>rpr*,*br*^*RNAi*^ adults after d9 ablation (*n* = 66 wings). *p* = 1.7 × 10^−53^, *p* = 8.5 × 10^−10^, and *p* = 1.7 × 10^−11^ (*rn*^*ts*^*>egr*,*yw* at d7 compared to *rn*^*ts*^*>egr*,*yw* at d9; *rn*^*ts*^*>egr*,*yw* at d7 compared to *rn*^*ts*^*>egr*,*chinmo*^*RNAi*^ at d7; and *rn*^*ts*^*>egr*,*yw* at d9 compared to *rn*^*ts*^*>egr*,*br*^*RNAi*^ at d9, respectively). Scale bars: 30 **μ**m. Underlying data for S1 Fig can be found in S1 Data. *br*, *broad*; d, day; DAPI, 4′,6-diamidino-2-phenylindole; Dcp-1, Death Caspase-1; *egr*, *eiger*; eL3, early L3; L3, third larval stage; RNAi, RNA interference; *rn*^*ts*^, *rotund-GAL4*, *tubulin-GAL80*^*thermo-sensitive*^; *rpr*, *reaper*; R0, beginning of the recovery period; Wg, Wingless; *yw*, *yellow*,*white*.
(PDF)

S2 Fig *br-Z2* and *br-Z3* are not expressed during L3 stages. (A) Br-Z2 (green) is absent in early L3 when Chinmo (magenta) is high and in late L3 when Chinmo is absent. Note that *br-Z2* is expressed in eL3 fat body cells [23]. (B) *br-Z3* (magenta) is not expressed during L3 stages. The specificity of the *br-Z3* antibody is demonstrated in GFP-marked *Flip-out* clone cells misexpressing *br-Z3*. Scale bars: 30 **μ**m. *br*, *broad*; eL3, early L3; GFP, green fluorescent protein; L3, third larval stage.
(PDF)

S3 Fig Chinmo is not post-transcriptionally regulated in wing imaginal discs. (A) The *mCherry*^*chinmoUTRs*^ transgene driven in the wing pouch by *nab-GAL4* leads to strong mCherry staining in wing discs of both early L3 and late L3. (B) *Flip-out* clones misexpressing *EcR*^*DN*^ exhibit strong anti-Chinmo staining (magenta, 58/62 clones, *n* = 8 discs) in late L3. (C) Misexpression of *EcR*^*DN*^ using *nab-GAL4* induces strong anti-Chinmo staining (magenta) in the wing pouch of late L3 larvae. (D) MARCM clones misexpressing *EcR*^*DN*^ exhibit decreased anti-Br staining (magenta, 7/7 clones, *n* = 3 discs) in late L3. (E) Misexpression of *EcR*^*DN*^ using *nab-GAL4* induces decreased anti-Br staining (magenta) in late L3. (F) Relative intensity of anti-Chinmo (magenta) and anti-Br (blue) staining in *EcR*^*RNAi*^ *Flip-out* clones represented in a log_10_ scale. Chinmo is down-regulated (*n* = 22 focal planes, 11 clones, 4 discs, m = 0.80 ± 0.041), whereas Br is up-regulated (*n* = 23 focal planes, 11 clones, 4 discs, m = 1.19 ± 0.044) in mid L3. (G) *EcR* (magenta) is expressed throughout L3 stages. (H) Anti-EcR staining remains constant in *chinmo*^*1*^ mutant MARCM clones (magenta, 26/26 clones, *n* = 5 discs). Scale bars: 30 **μ**m. Underlying data for S3 Fig can be found in S1 Data. *br*, *broad*; *EcR*^*DN*^, dominant negative form of ecdysone receptor; eL3, early L3; *FO*, *Flip-*out; GFP, green fluorescent protein; L3, third larval stage; MARCM, Mosaic Analysis with a Repressible Cell Marker; RNAi, RNA interference.
(PDF)

S4 Fig Cross-repressive interactions of Chinmo and Br. *GAL4* expression and *Flip-out* clones are marked with GFP and outlined in yellow. (A–B) Misexpression of *chinmo* using *en-GAL4* (A) and *nab-GAL4* (B) leads to *br* repression (magenta) during late L3. *UAS-p35* is coexpressed to inhibit apoptosis induced upon wide chinmo misexpression in late L3. (C) Misexpression of *br-Z1* using *nab-GAL4* leads to strong *chinmo* repression (magenta) during mid L3. (D–E) Misexpression of *br*^*RNAi*^ using *en-GAL4* (E) and *nab-GAL4* (F) triggers ectopic *chinmo* expression (magenta) in late L3. (F) Down-regulation of Br by misexpressing *br*^*RNAi*^ using *nab-GAL4* leads to ectopic *chinmo-lacZ* expression in the wing pouch of late L3 larvae. (G) Misexpression of *br-Z2* in *Flip-out* clones leads to strong cell lethality, as shown by Dcp-1 staining and pyknotic cells revealed with the DAPI staining. (H, I) Misexpression of *br-Z3* (H) and *br-Z4* (I) in mid L3 reduces *chinmo* expression. Scale bars: 30 **μ**m. *br*, *broad*; DAPI, 4′,6-diamidino-2-phenylindole; Dcp-1, Death Caspase-1; *en*, *engrailed*; GFP, green fluorescent protein;; L3, third larval stage; RNAi, RNA interference; UAS, Upstream Activating Sequence.
(PDF)

S5 Fig Chinmo maintenance prevents ecdysone-mediated differentiation. *GAL4* expression and MARCM clones are marked with GFP and outlined in yellow. (A) Misexpression of *EcR*^*DN*^ using *nab-GAL4* prevents Sens (magenta) and Cut (blue) expression. (B–C) Misexpression of *chinmo* using *nab-GAL4* (B) or *en-GAL4* (C) prevents Sens (magenta) and Cut (blue) expression. *UAS-p35* is expressed at the same time to inhibit apoptosis induced when *chinmo* is widely misexpressed. (D) Cut (magenta) is not ectopically expressed in *chinmo* mutant MARCM clones in early L3 before the CW. Scale bars: 30 **μ**m. CW, critical weight; *EcR*^*DN*^, dominant negative form of ecdysone receptor; eL3, early L3; *en*, *engrailed*; L3, third larval stage; MARCM, Mosaic Analysis with a Repressible Cell Marker; *sens*, *senseless*; UAS, Upstream Activating Sequence.
(PDF)

S6 Fig Wg and Chinmo intensities decrease when *br-Z1* is misexpressed during ablation process. (A) Relative anti-Wg staining intensity in the wing pouch at R0 upon d7 ablation in *rn*^*ts*^*>egr* larvae (*n* = 13 wing discs, m = 2.57 ± 0.13) and *rn*^*ts*^*>egr*,*br-Z1* larvae (*n* = 11 wing discs, m = 2.08 ± 0.10). *p* = 0.040. (B) Relative anti-Chinmo staining intensity in the wing pouch at R0 upon d7 ablation in *rn*^*ts*^*>egr* larvae (*n* = 13 wing discs, m = 2.01 ± 0.09) and *rn*^*ts*^*>egr*,*br-Z1* larvae (*n* = 12 wing discs, m = 1.84 ± 0.07). *p* = 0.225. Scale bars: 30 **μ**m. Underlying data for S6 Fig can be found in S1 Data. *br*, *broad*; d, day; *egr*, *eiger*; *rn*^*ts*^, *rotund-GAL4*, *tubulin-GAL80*^*thermo-sensitive*^; R0, beginning of the recovery period; Wg, Wingless.
(PDF)

S7 Fig Chinmo and Br govern the regenerative potential of wing epithelium. (A) Anti-Pdm1/Nub (green) and DAPI (magenta) wing disc stainings at R0 after d9 ablation in various genetic conditions, showing that the wing pouch appeared less folded when *EcR*^*DN*^, *chinmo*, and *br*^*RNAi*^ were misexpressed. (B) Relative anti-Wg staining intensity in the wing pouch at R0 upon d9 ablation in *rn*^*ts*^*>egr* larvae (*n* = 10 wing discs, m = 2.01 ± 0.12), *rn*^*ts*^*>egr*,*EcR*^*DN*^ larvae (*n* = 9 wing discs, m = 2.20 ± 0.07), *rn*^*ts*^*>egr*,*EcR*^*DN*^,*chinmo*^*RNAi*^ larvae (*n* = 8 wing discs, m = 1.78 ± 0.11), *rn*^*ts*^*>egr*,*chinmo* (*n* = 11 wing discs, m = 2.15 ± 0.12), and *rn*^*ts*^*>egr*,*br*^*RNAi*^ (*n* = 10 wing discs, m = 2.62 ± 0.18). *p* = 0.112, *p* = 0.011, *p* = 0.605, and *p* = 0.005 (*rn*^*ts*^*>egr* compared to *rn*^*ts*^*>egr*,*EcR*^*DN*^; *rn*^*ts*^*>egr*,*EcR*^*DN*^ compared to *rn*^*ts*^*>egr*,*EcR*^*DN*^,*chinmo*^*RNAi*^; *rn*^*ts*^*>egr* compared to *rn*^*ts*^*>egr*,*chinmo*; and *rn*^*ts*^*>egr* compared to *rn*^*ts*^*>egr*,*br*^*RNAi*^, respectively). (C) Relative anti-Chinmo staining intensity in the wing pouch at R0 upon d9 ablation in *rn*^*ts*^*>egr* larvae (*n* = 12 wing discs, m = 1.65 ± 0.06), *rn*^*ts*^*>egr*,*EcR*^*DN*^ larvae (*n* = 9 wing discs, m = 2.78 ± 0.13), *rn*^*ts*^*>egr*,*EcR*^*DN*^,*chinmo*^*RNAi*^ larvae (*n* = 8 wing discs, m = 1.71 ± 0.13), *rn*^*ts*^*>egr*,*chinmo* (*n* = 10 wing discs, m = 5.24 ± 0.50), and *rn*^*ts*^*>egr*,*br*^*RNAi*^ larvae (*n* = 10 wing discs, m = 3.57 ± 0.26). *p* = 6.8 × 10^−6^, *p* = 8.2 × 10^−5^, *p* = 3.1 × 10^−6^, and *p* = 8.7 × 10^−5^ (*rn*^*ts*^*>egr* compared to *rn*^*ts*^*>egr*,*EcR*^*DN*^; *rn*^*ts*^*>egr*,*EcR*^*DN*^ compared to *rn*^*ts*^*>egr*,*EcR*^*DN*^,*chinmo*^*RNAi*^; *rn*^*ts*^*>egr* compared to *rn*^*ts*^*>egr*,*chinmo*; and *rn*^*ts*^*>egr* compared to *rn*^*ts*^*>egr*,*br*^*RNAi*^, respectively). (D) Misexpression of *EcR*^*DN*^ in the posterior compartment of undamaged late L3 wing disc using *en-GAL4* does not induce ectopic *wg* expression. (E) Volume of anti-Dcp-1 staining over total wing disc volume at R0 upon d9 ablation in *rn*^*ts*^*>egr* larvae (*n* = 7 wing discs, m = 0.119 ± 0.009), *rn*^*ts*^*>egr*,*EcR*^*DN*^ larvae (*n* = 7 wing discs, m = 0.046 ± 0.007), *rn*^*ts*^*>egr*,*EcR*^*DN*^,*chinmo*^*RNAi*^ larvae (*n* = 7 wing discs, m = 0.047 ± 0.005), *rn*^*ts*^*>egr*,*chinmo* larvae (*n* = 8 wing discs, m = 0.128 ± 0.009), and *rn*^*ts*^*>egr*,*br*^*RNAi*^ larvae (*n* = 9 wing discs, m = 0.091 ± 0.014). *p* = 5.8 × 10^−4^, *p* = 1, *p* = 0.612, and *p* = 0.091 (*rn*^*ts*^*>egr* compared to *rn*^*ts*^*>egr*,*EcR*^*DN*^; *rn*^*ts*^*>egr*,*EcR*^*DN*^ compared to *rn*^*ts*^*>egr*,*EcR*^*DN*^,*chinmo*^*RNAi*^; *rn*^*ts*^*>egr* compared to *rn*^*ts*^*>egr*,*chinmo*; and *rn*^*ts*^*>egr* compared to *rn*^*ts*^*>egr*,*br*^*RNAi*^, respectively). Scale bars: 30 **μ**m. Underlying data for S7 Fig can be found in S1 Data. *br*, *broad*; d, day; DAPI, 4′,6-diamidino-2-phenylindole; Dcp-1, Death Caspase-1; *EcR*^*DN*^, dominant negative form of ecdysone receptor; *egr*, *eiger*; *en*, *engrailed*; L3, third larval stage; Nub, Nubbin; Pdm1, POU domain protein 1; RNAi, RNA interference; *rn*^*ts*^, *rotund-GAL4*, *tubulin-GAL80*^*thermo-sensitive*^; R0, beginning of the recovery period; vol, volume; Wg, Wingless.
(PDF)

S1 Data Values used to generate graphs. (XLSX)

## Abbreviations

*br*: *broad*
CNS: central nervous system
CW: critical weight
d: day
Dcp-1: Death Caspase-1
DGRC: *Drosophila* Genetic Resource Consortium
Dilp8: *Drosophila* Insulin-like peptide 8
DSHB: Developmental Studies Hybridoma Bank
*EcR^DN^*: dominant negative form of ecdysone receptor
*egr*: *eiger*
*en*: *engrailed*
*FO*: *Flip-out*
GFP: green fluorescent protein
JAK/STAT: Janus Kinase/Signal Transducers and Activators of Transcription
JNK: c-Jun N-terminal kinase
L3: third larval stage
m ± SEM: mean ± standard error of the mean
MARCM: Mosaic Analysis with a Repressible Cell Marker
Mmp1: Matrix metalloprotease 1
NB: neuroblast
PcG: Polycomb-Group
Pdm1/Nub: POU domain Protein 1/Nubbin
PICsL: Shared Imaging Platform of Luminy Campus
RNAi: RNA interference
*rn^ts^*: *rotund-GAL4*,*tubulin-GAL80*^*thermo-sensitive*^
ROS: reactive oxygen species
*rpr*: *reaper*
R0: beginning of the recovery period
*sens*: *senseless*
TRiP: Transgenic RNAi Project
UAS: Upstream Activating Sequence
vol: volume
Wg: Wingless
*yw*: *yellow*,*white*
ZBTB: Broad-complex/Tramtrack/Bric-à-brac Zinc-finger
20-HE: 20-hydroxyecdysone
