# Supplementary figures and images for: Developmental regulation of regenerative potential in Drosophila by ecdysone through a bistable loop of ZBTB transcription factors
Source: PLoS Biol. 2019 Feb 11;17(2):e3000149. doi: 10.1371/journal.pbio.3000149 (PMC6386533; doi:10.1371/journal.pbio.3000149)

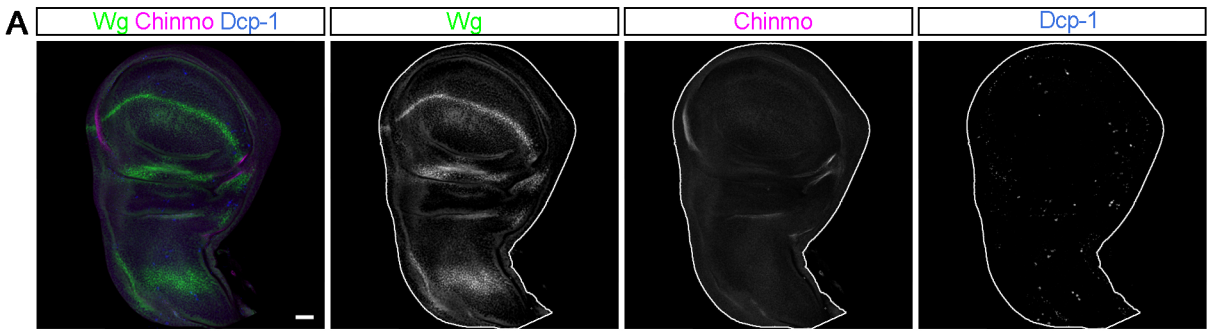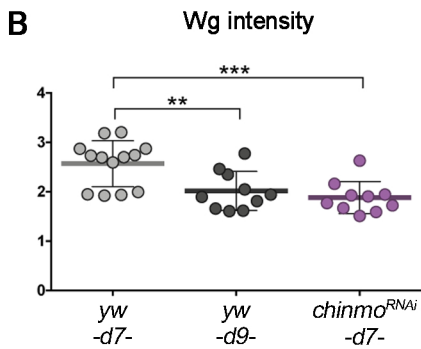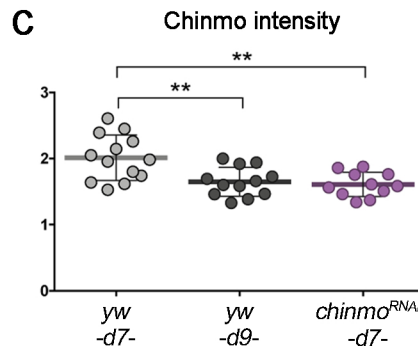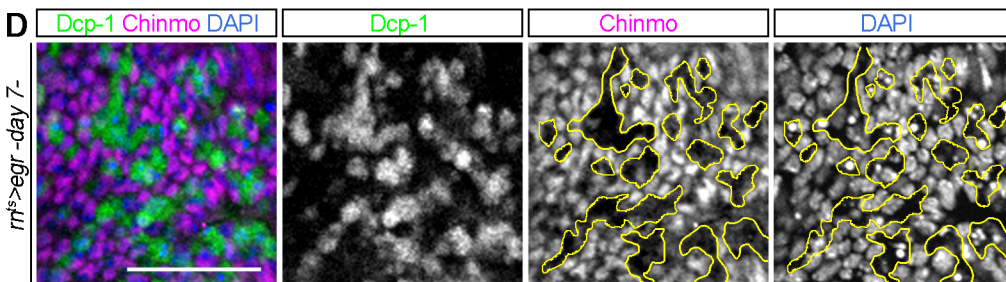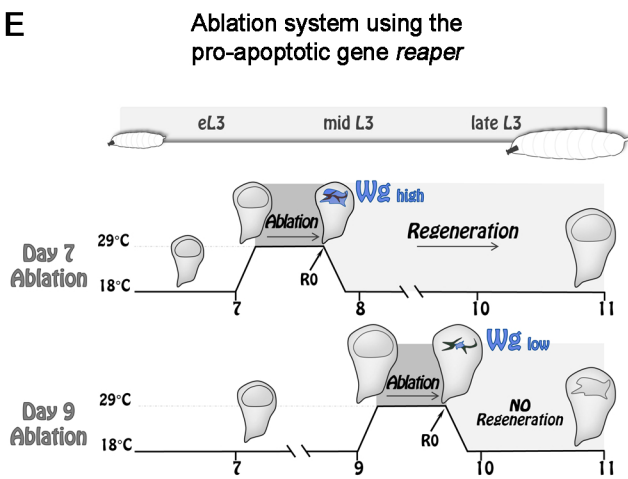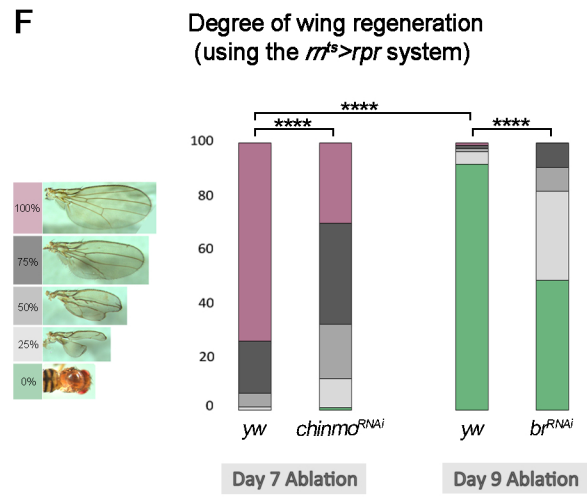

Supplement: S1 Fig — (A) Anti-Wg (green), anti-Chinmo (magenta), and anti-Dcp-1 (blue) stainings in an undamaged late L3 wing disc. (B) Relative anti-Wg staining intensity in the wing pouch of rnts>egr larvae at R0 after d7 ablation (n = 13 wing discs, m = 2.57 ± 0.13), of rnts>egr at R0 after d9 ablation (n = 10 wing discs, m = 2.01 ± 0.12), and of rnts>egr,chinmoRNAi larvae at R0 after d7 ablation (n = 10 wing discs, m = 1.88 ± 0.10). p = 0.0080 and p = 0.0005 (rnts>egr,yw at d7 compared to rnts>egr,yw at d9 and rnts>egr,yw at d7 compared to rnts>egr,chinmoRNAi at d7, respectively). (C) Relative anti-Chinmo staining intensity in the wing pouch of rnts>egr larvae at R0 after d7 ablation (n = 13 wing discs, m = 2.01 ± 0.09), of rnts>egr larvae at R0 after d9 ablation (n = 12 wing discs, m = 1.65 ± 0.06), and of rnts>egr,chinmoRNAi larvae at R0 after d7 ablation (n = 11 wing discs, m = 1.61 ± 0.18). p = 0.0055 and p = 0.0025 (rnts>egr,yw at d7 compared to rnts>egr,yw at d9 and rnts>egr,yw at d7 compared to rnts>egr,chinmoRNAi at d7, respectively). (D) Chinmo (magenta) is low in dying cells outlined in yellow, marked by Dcp-1 staining (green) and pyknotic nuclei seen with DAPI staining (blue). (E) Schematic representation of the rnts>rpr ablation system used to induce wing pouch ablation. Strong wg expression at R0 is observed in response to damage when ablation is initiated at d7 for 20 hours. wg expression is drastically reduced when ablation is initiated at d9. From [9]. (F) Examples of wing size scores are shown. Distribution of wing size from rnts>rpr,yw adults after d7 ablation (n = 1,217 wings); rnts>rpr,chinmoRNAi adults after d7 ablation (n = 186 wings); rnts>rpr,yw adults after d9 ablation (n = 185 wings); and rnts>rpr,brRNAi adults after d9 ablation (n = 66 wings). p = 1.7 × 10^−53^, p = 8.5 × 10^−10^, and p = 1.7 × 10^−11^ (rnts>egr,yw at d7 compared to rnts>egr,yw at d9; rnts>egr,yw at d7 compared to rnts>egr,chinmoRNAi at d7; and rnts>egr,yw at d9 compared to rnts>egr,brRNAi at d9 [file pbio.3000149.s001.pdf]

yw

A

eL3

late L3

DNA Br-Z2 Chinmo

Br-Z2

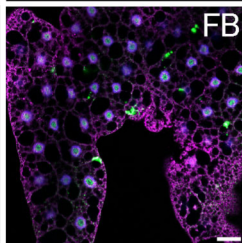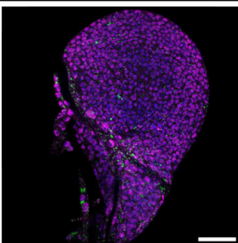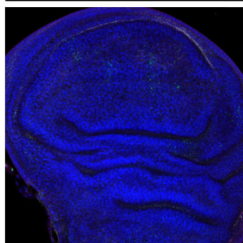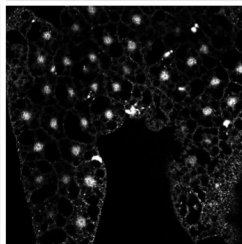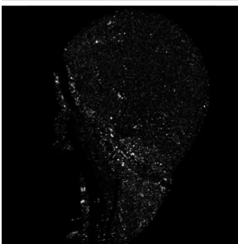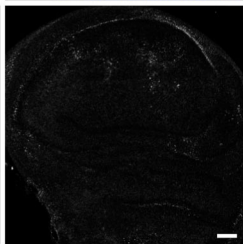

*FO>GFP, br-Z3*

B

eL3

midL3

late L3

DNA GFP Br-Z3

Br-Z3

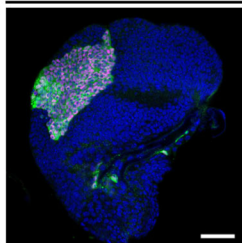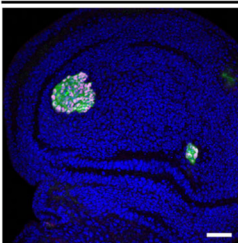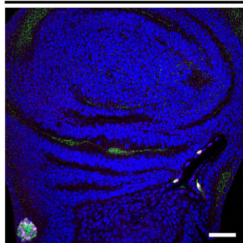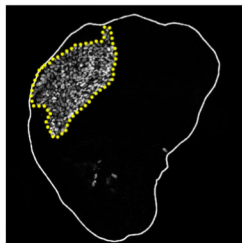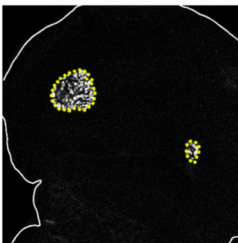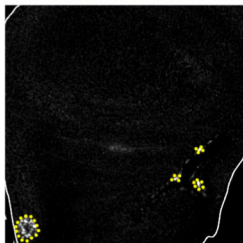

Supplement: S2 Fig — (A) Br-Z2 (green) is absent in early L3 when Chinmo (magenta) is high and in late L3 when Chinmo is absent. Note that br-Z2 is expressed in eL3 fat body cells [23]. (B) br-Z3 (magenta) is not expressed during L3 stages. The specificity of the br-Z3 antibody is demonstrated in GFP-marked Flip-out clone cells misexpressing br-Z3. Scale bars: 30 μm. br, broad; eL3, early L3; GFP, green fluorescent protein; L3, third larval stage. (PDF) [file pbio.3000149.s002.pdf]

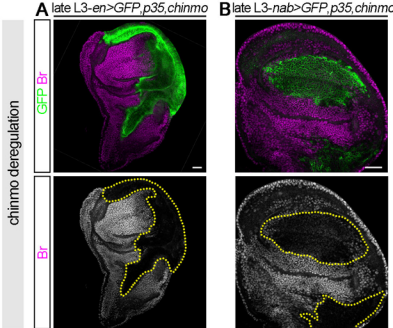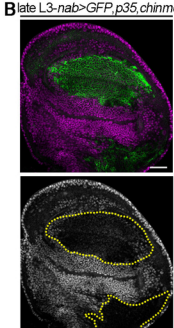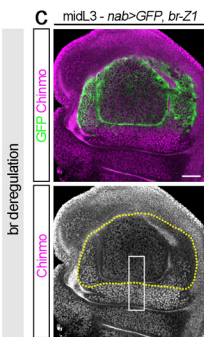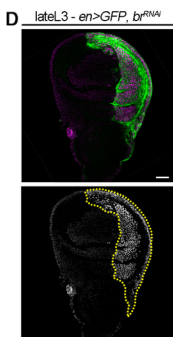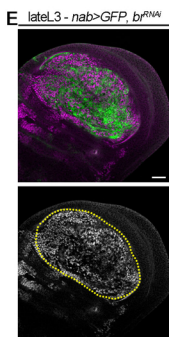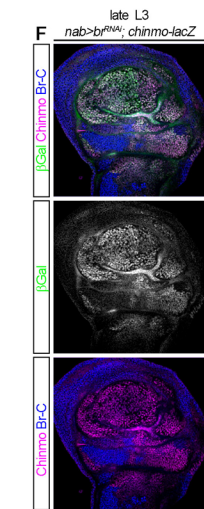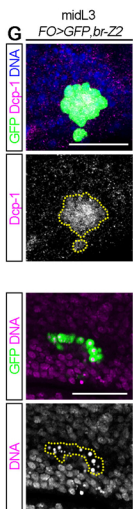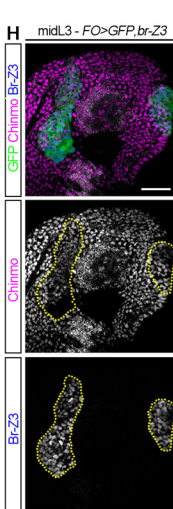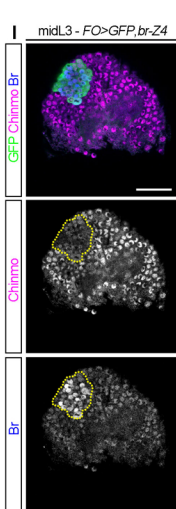

Supplement: S4 Fig — GAL4 expression and Flip-out clones are marked with GFP and outlined in yellow. (A–B) Misexpression of chinmo using en-GAL4 (A) and nab-GAL4 (B) leads to br repression (magenta) during late L3. UAS-p35 is coexpressed to inhibit apoptosis induced upon wide chinmo misexpression in late L3. (C) Misexpression of br-Z1 using nab-GAL4 leads to strong chinmo repression (magenta) during mid L3. (D–E) Misexpression of brRNAi using en-GAL4 (E) and nab-GAL4 (F) triggers ectopic chinmo expression (magenta) in late L3. (F) Down-regulation of Br by misexpressing brRNAi using nab-GAL4 leads to ectopic chinmo-lacZ expression in the wing pouch of late L3 larvae. (G) Misexpression of br-Z2 in Flip-out clones leads to strong cell lethality, as shown by Dcp-1 staining and pyknotic cells revealed with the DAPI staining. (H, I) Misexpression of br-Z3 (H) and br-Z4 (I) in mid L3 reduces chinmo expression. Scale bars: 30 μm. br, broad; DAPI, 4′,6-diamidino-2-phenylindole; Dcp-1, Death Caspase-1; en, engrailed; GFP, green fluorescent protein;; L3, third larval stage; RNAi, RNA interference; UAS, Upstream Activating Sequence. (PDF) [file pbio.3000149.s004.pdf]

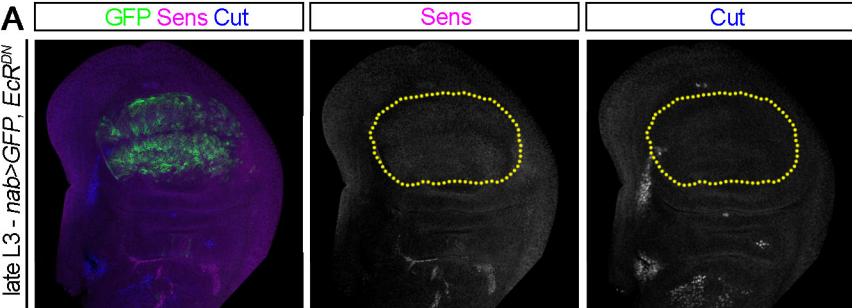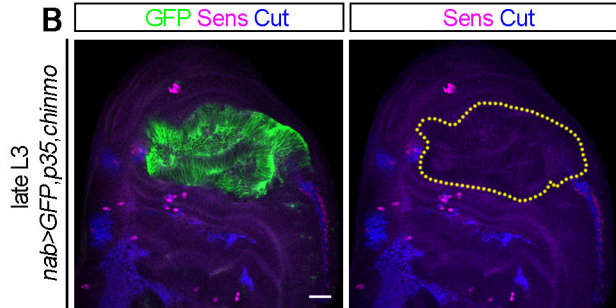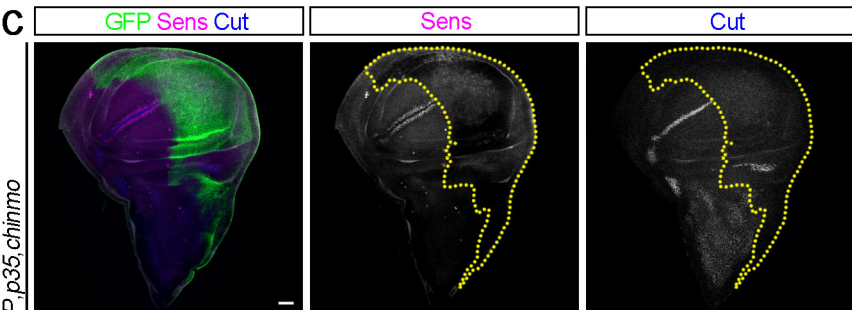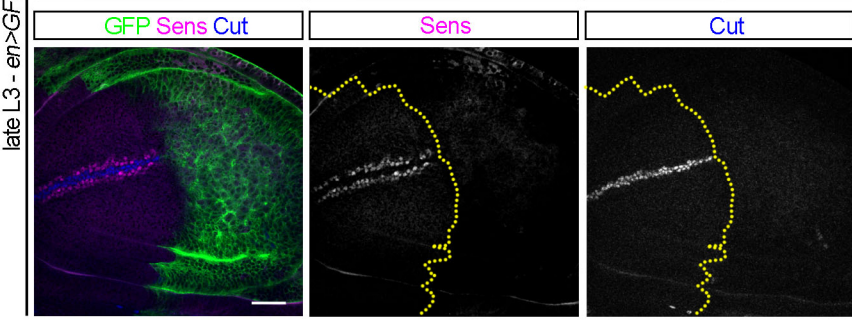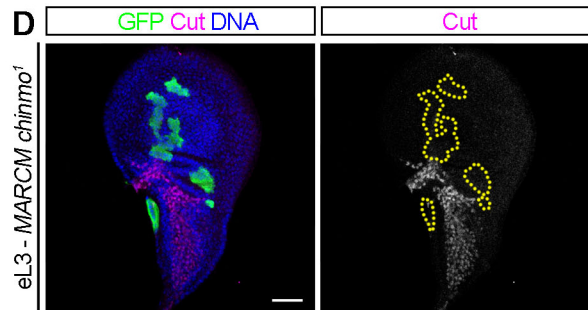

Supplement: S5 Fig — GAL4 expression and MARCM clones are marked with GFP and outlined in yellow. (A) Misexpression of EcRDN using nab-GAL4 prevents Sens (magenta) and Cut (blue) expression. (B–C) Misexpression of chinmo using nab-GAL4 (B) or en-GAL4 (C) prevents Sens (magenta) and Cut (blue) expression. UAS-p35 is expressed at the same time to inhibit apoptosis induced when chinmo is widely misexpressed. (D) Cut (magenta) is not ectopically expressed in chinmo mutant MARCM clones in early L3 before the CW. Scale bars: 30 μm. CW, critical weight; EcRDN, dominant negative form of ecdysone receptor; eL3, early L3; en, engrailed; L3, third larval stage; MARCM, Mosaic Analysis with a Repressible Cell Marker; sens, senseless; UAS, Upstream Activating Sequence. (PDF) [file pbio.3000149.s005.pdf]

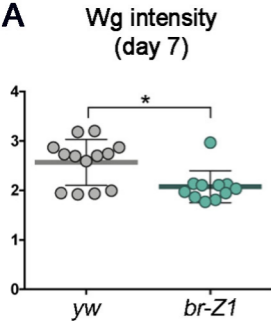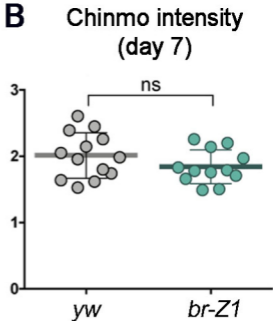

Supplement: S6 Fig — (A) Relative anti-Wg staining intensity in the wing pouch at R0 upon d7 ablation in rnts>egr larvae (n = 13 wing discs, m = 2.57 ± 0.13) and rnts>egr,br-Z1 larvae (n = 11 wing discs, m = 2.08 ± 0.10). p = 0.040. (B) Relative anti-Chinmo staining intensity in the wing pouch at R0 upon d7 ablation in rnts>egr larvae (n = 13 wing discs, m = 2.01 ± 0.09) and rnts>egr,br-Z1 larvae (n = 12 wing discs, m = 1.84 ± 0.07). p = 0.225. Scale bars: 30 μm. Underlying data for S6 Fig can be found in S1 Data. br, broad; d, day; egr, eiger; rnts, rotund-GAL4, tubulin-GAL80thermo-sensitive; R0, beginning of the recovery period; Wg, Wingless. (PDF) [file pbio.3000149.s006.pdf]

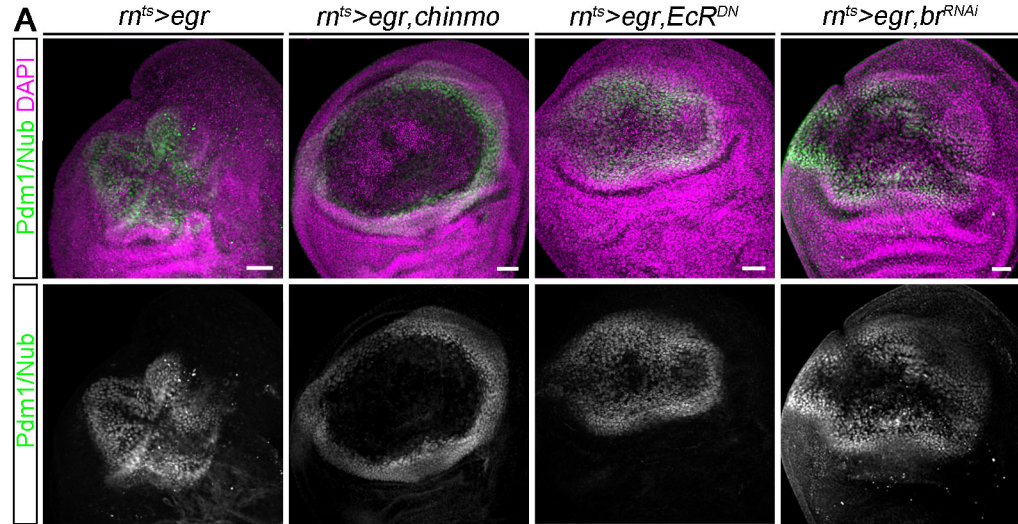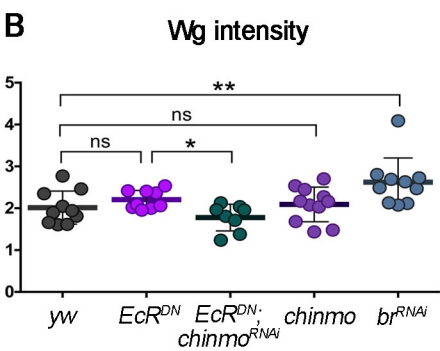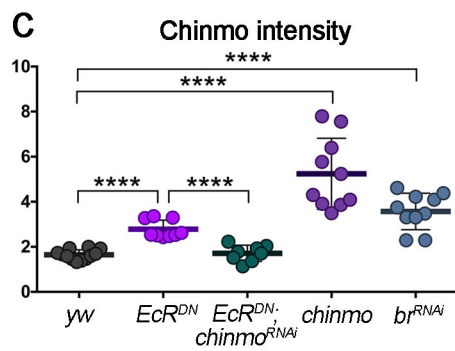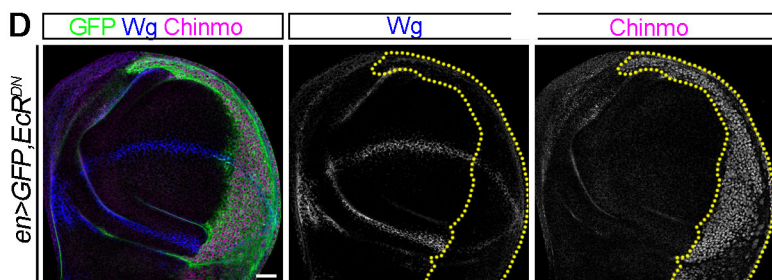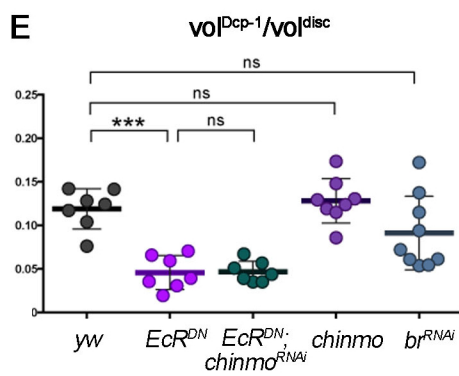

Supplement: S7 Fig — (A) Anti-Pdm1/Nub (green) and DAPI (magenta) wing disc stainings at R0 after d9 ablation in various genetic conditions, showing that the wing pouch appeared less folded when EcRDN, chinmo, and brRNAi were misexpressed. (B) Relative anti-Wg staining intensity in the wing pouch at R0 upon d9 ablation in rnts>egr larvae (n = 10 wing discs, m = 2.01 ± 0.12), rnts>egr,EcRDN larvae (n = 9 wing discs, m = 2.20 ± 0.07), rnts>egr,EcRDN,chinmoRNAi larvae (n = 8 wing discs, m = 1.78 ± 0.11), rnts>egr,chinmo (n = 11 wing discs, m = 2.15 ± 0.12), and rnts>egr,brRNAi (n = 10 wing discs, m = 2.62 ± 0.18). p = 0.112, p = 0.011, p = 0.605, and p = 0.005 (rnts>egr compared to rnts>egr,EcRDN; rnts>egr,EcRDN compared to rnts>egr,EcRDN,chinmoRNAi; rnts>egr compared to rnts>egr,chinmo; and rnts>egr compared to rnts>egr,brRNAi, respectively). (C) Relative anti-Chinmo staining intensity in the wing pouch at R0 upon d9 ablation in rnts>egr larvae (n = 12 wing discs, m = 1.65 ± 0.06), rnts>egr,EcRDN larvae (n = 9 wing discs, m = 2.78 ± 0.13), rnts>egr,EcRDN,chinmoRNAi larvae (n = 8 wing discs, m = 1.71 ± 0.13), rnts>egr,chinmo (n = 10 wing discs, m = 5.24 ± 0.50), and rnts>egr,brRNAi larvae (n = 10 wing discs, m = 3.57 ± 0.26). p = 6.8 × 10^−6^, p = 8.2 × 10^−5^, p = 3.1 × 10^−6^, and p = 8.7 × 10^−5^ (rnts>egr compared to rnts>egr,EcRDN; rnts>egr,EcRDN compared to rnts>egr,EcRDN,chinmoRNAi; rnts>egr compared to rnts>egr,chinmo; and rnts>egr compared to rnts>egr,brRNAi, respectively). (D) Misexpression of EcRDN in the posterior compartment of undamaged late L3 wing disc using en-GAL4 does not induce ectopic wg expression. (E) Volume of anti-Dcp-1 staining over total wing disc volume at R0 upon d9 ablation in rnts>egr larvae (n = 7 wing discs, m = 0.119 ± 0.009), rnts>egr,EcRDN larvae (n = 7 wing discs, m = 0.046 ± 0.007), rnts>egr,EcRDN,chinmoRNAi larvae (n = 7 wing discs, m = 0.047 ± 0.005), rnts>egr,chinmo larvae (n = 8 wing discs, m = 0.128 ± 0.009), and rnts>egr,brRNAi larvae (n = 9 wing discs, [file pbio.3000149.s007.pdf]
