# Supplementary material for: Developmental regulation of regenerative potential in Drosophila by ecdysone through a bistable loop of ZBTB transcription factors
Source: PLoS Biol. 2019 Feb 11;17(2):e3000149. doi: 10.1371/journal.pbio.3000149 (PMC6386533; doi:10.1371/journal.pbio.3000149)
Supplement: S3 Fig — (A) The mCherrychinmoUTRs transgene driven in the wing pouch by nab-GAL4 leads to strong mCherry staining in wing discs of both early L3 and late L3. (B) Flip-out clones misexpressing EcRDN exhibit strong anti-Chinmo staining (magenta, 58/62 clones, n = 8 discs) in late L3. (C) Misexpression of EcRDN using nab-GAL4 induces strong anti-Chinmo staining (magenta) in the wing pouch of late L3 larvae. (D) MARCM clones misexpressing EcRDN exhibit decreased anti-Br staining (magenta, 7/7 clones, n = 3 discs) in late L3. (E) Misexpression of EcRDN using nab-GAL4 induces decreased anti-Br staining (magenta) in late L3. (F) Relative intensity of anti-Chinmo (magenta) and anti-Br (blue) staining in EcRRNAi Flip-out clones represented in a log10 scale. Chinmo is down-regulated (n = 22 focal planes, 11 clones, 4 discs, m = 0.80 ± 0.041), whereas Br is up-regulated (n = 23 focal planes, 11 clones, 4 discs, m = 1.19 ± 0.044) in mid L3. (G) EcR (magenta) is expressed throughout L3 stages. (H) Anti-EcR staining remains constant in chinmo1 mutant MARCM clones (magenta, 26/26 clones, n = 5 discs). Scale bars: 30 μm. Underlying data for S3 Fig can be found in S1 Data. br, broad; EcRDN, dominant negative form of ecdysone receptor; eL3, early L3; FO, Flip-out; GFP, green fluorescent protein; L3, third larval stage; MARCM, Mosaic Analysis with a Repressible Cell Marker; RNAi, RNA interference. (PDF) [file pbio.3000149.s003.pdf]

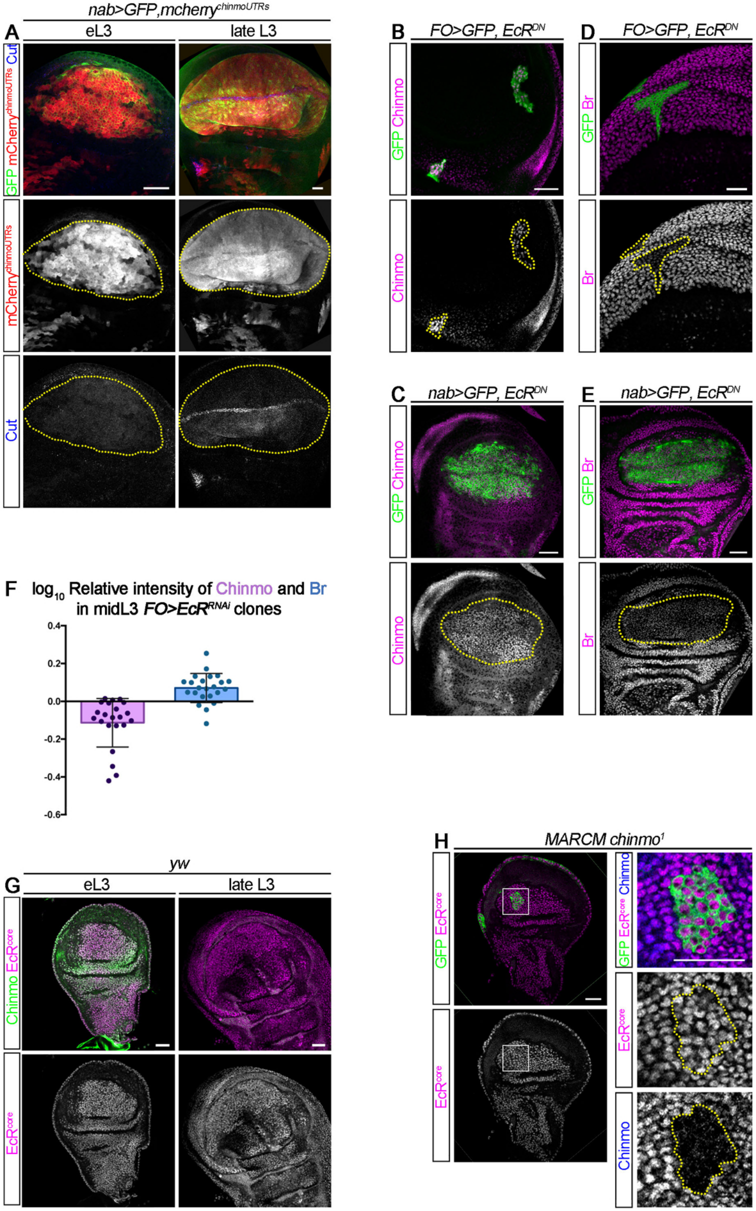

nab>GFP,mcherry^chinmoUTRs
A
eL3
late L3
GFP mCherry^chinmoUTRs Cut
mCherry^chinmoUTRs
Cut
B
FO>GFP, EcR^DN
GFP Chinmo
Chinmo
D
FO>GFP, EcR^DN
GFP Br
Br
C
nab>GFP, EcR^DN
GFP Chinmo
Chinmo
E
nab>GFP, EcR^DN
GFP Br
Br
F
log10 Relative intensity of Chinmo and Br in midL3 FO>EcR^RNAi clones
0.4
0.2
0.0
-0.2
-0.4
-0.6
G
yw
eL3
late L3
Chinmo EcR^core
EcR^core
H
MARCM chinmo^1
GFP EcR^core
EcR^core
GFP EcR^core Chinmo
EcR^core
Chinmo
